# Competitive and sequence reactions of typical hydrocarbon molecules in diesel fraction hydrocracking – a theoretical study by DFT calculations

**DOI:** 10.1039/d1ra09246d

**Published:** 2022-07-08

**Authors:** Ji-Feng Wang, Si-Jia Ding, Shao-Zhong Peng, Zhan-Lin Yang, Yan-Ze Du

**Affiliations:** Dalian Research Institute of Petroleum and Petrochemicals, SINOPEC Dalian 116041 China duyanze.fshy@sinopec.com

## Abstract

The molecular structures of hydrocarbon molecules determine the competitive and sequence reactions in the diesel hydrocracking process. In this study, the hydrocracking reactions of typical hydrocarbons with various saturation degrees and molecular weights in diesel fractions synergistically catalyzed by the Ni–Mo–S nanocluster and Al–Si FAU zeolite are investigated. The results show that the two major rate-controlling steps in saturated hydrocarbon hydrocracking are dehydrogenation on the Ni–Mo–S active sites and the cracking of the C–C bonds on the FAU zeolite acid center. Moreover, the major rate-controlling step in cracking the cycloalkyl aromatic hydrocarbons is the protonation of the aromatic ring. Moreover, the aromatic hydrocarbons presented an apparent advantage in competitive adsorption on the Ni–Mo–S active sites, whereas hydrocarbons with higher molecular weights demonstrated a moderate adsorption advantage on both Ni–Mo–S active sites and FAU zeolite acid centers.

## Abstract 2

The molecular structures of hydrocarbon molecules determine the competitive and sequence reactions in the diesel hydrocracking process.

## Graphical abstract

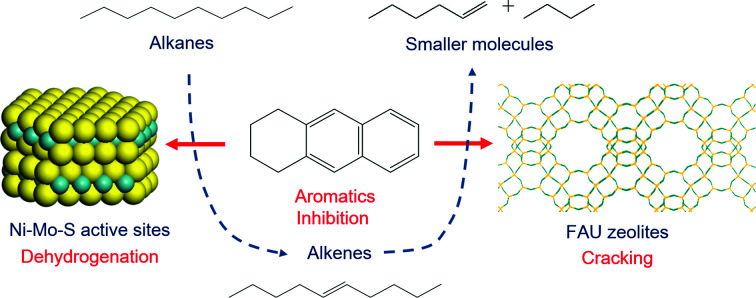

## 1 Introduction

Restricted by the commitments toward emission reduction, the petrochemical industry is facing the pressing issue of excessive vehicular diesel production. Hydrocracking is an effective technology to consume diesel, particularly inferior quality diesel, to produce aviation kerosene and chemical raw materials.^1–5^ Compared with conventional gas oil hydrocracking, diesel hydrocracking is operated under higher liquid space velocity but lower temperature and hydrogen pressure. Moreover, diesel hydrocracking requires more accurate control over the conversion reactions to achieve the expected product distribution.^6–8^ The difficulties in the development of an ideal hydrocracking catalyst do not only include the composition and distributional control of the active centers for hydrogenation and cracking to achieve optimal spatial and functional coordination, but also the fine structure and surface adjustments in the atomic scale to regulate the reaction sequences of the complex feedstock.^9–13^ Therefore, a deep understanding of the competitive and sequence reactions among these various reactants is fundamental of catalytic research and development.

The compositional complexity of the feedstock includes the differences in the functional groups and molecular weights. The structural differences between the hydrocarbons determine the reaction routes in two ways. On the one hand, unsaturated hydrocarbons, including olefins and aromatics, can be protonated directly on the B-acid centers of the hydrocracking catalyst, whereas saturated hydrocarbons, including the alkanes and cycloalkanes, need to be dehydrogenated on the metal active sites before protonation.^14,15^ On the other hand, the stability of the carbonium ion on the acid sites also affects the cracking ability of the C–C bond and the probability for multiple cracking routes.^16–18^

DFT calculation is a widely used theoretical method to study the mechanism and structure–activity relationship in catalysis and the petrochemical industry.^19–24^ In the field of hydrocracking research, the hydrogenation mechanism and elementary reactions of hydrocarbons on the metal sulfide active sites have been calculated.^25^ In addition, the carbocation pathways of some small hydrocarbons on zeolites, including ZSM, FER and FAU, have also been reported.^26–29^ These preliminary studies clarify the key elementary reactions of the hydrocracking process. In this study, DFT calculations are used to study the key hydrocracking steps of typical diesel molecules on the Ni–Mo–S metal active sites and Al–Si FAU acid sites. Therefore, the reaction characteristics of diesel feedstock and competition among its components are expected to become more distinct and provide valuable references for the improvement of diesel hydrocracking catalysts.

## 2 Modeling and computational methods

### 2.1 Modeling

**Fig. 1:**
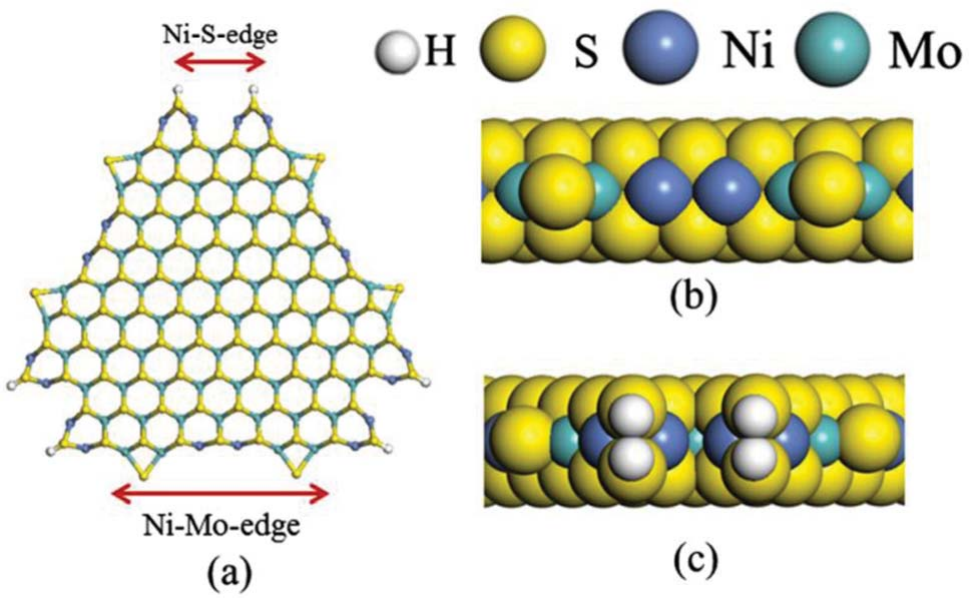
Ni–Mo–S model.

**Fig. 2:**
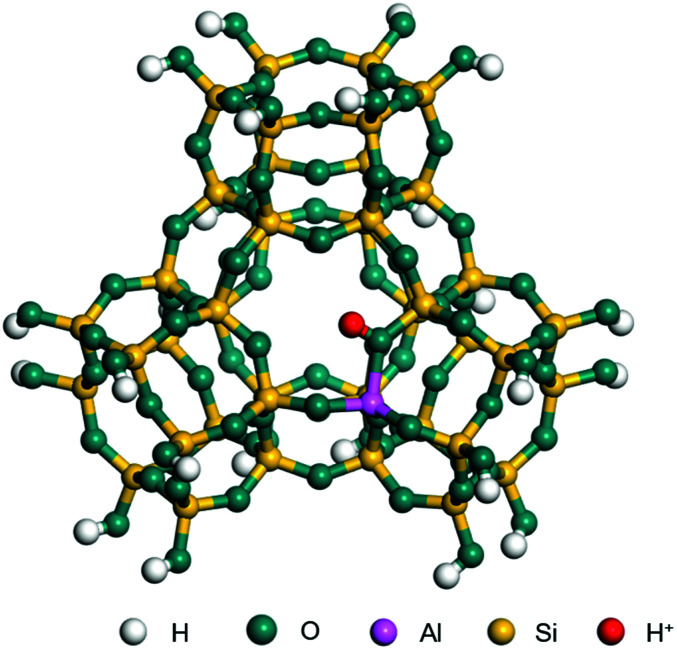
FAU model.

The dehydrogenation of alkanes and cycloalkanes was performed on the Ni–Mo-edge of the Ni–Mo–S bimetal active sites, and the calculation model was established, as shown in Fig. 1. On the Ni–Mo-edge, the nickel coverage was 50% percent. The Ni atom was in square-planar coordination with four S atoms, and the normal direction of the square plane was exposed without the S atoms.^21,25,30^ The hydrocracking of olefins and aromatics was performed on the zeolite model extracted from the silica FAU structured cell (denoted as FAU-Al). The zeolite model consisted of four β-cages and one super-cage.^31–33^ Each outer oxygen atom was bonded with one hydrogen atom to lower the surface energy and maintain structural stability. One silica atom on the interface between the β-cage and one super-cage was substituted by an aluminum atom. The adjacent oxygen atom was attached with one additional hydrogen to achieve charge balance (Fig. 2). This area could be considered as one strong B-acid center of zeolite and the location at which the cracking reaction would take place.^34,35^

### 2.2 Computational methods

The calculations were performed using the DMol^3^ code with numerical atomic functions. The exchange-correlation function was the revised Perdew–Burke–Ernzerhof function belonging to the general gradient approximation rung,^36,37^ and the basis set was double numerical plus polarization (DNP).^38^ This calculation strategy was adopted to balance the calculation speed and the accuracy, which is suitable for the preliminary quantitative calculation of the non-periodic structures. To analyze the transition state, the electron spin was set to open shell mode. To reduce the energy error caused by the basis set, the BSSE correction was used in the calculation of adsorption. Specifically, the basis set group 1 was the adsorbing hydrocarbon and group 2 was the metal active site or zeolite. For thermodynamics calculations, the temperature was set to 640 K, the hydrogen pressure was set to 10.0 MPa, and the partial pressure of the hydrocarbons was set to 0.1 MPa. The thermodynamic theory was based on the work by Masel,^39^ and the specific use in hydrogenation calculations was adopted from another work:^22^ The adsorbed molecule may still retain some translational or rotational degrees of freedom upon adsorption. In this study, the thermodynamic rotational and translational entropies were balanced by the coefficient 1/3 with respect to the gas phase (based on the ESI of ref. 22 the third hypothesis in ESI-2).

The complete linear synchronous transit (LST) and quadratic synchronous transit (QST) methods were used to find the transition state, while the nudged elastic band (NEB) method was used to confirm the transition state. The D2 correction method was used to calculate the dispersion force.^40,41^ Other calculation details and parameters are listed in Table 1.

**Table 1:** Detailed calculation parameters

Calculation details		Parameters
Electronic treatment	Orbital cut off	5.0 Å
	Thermal smearing	5 × 10^−4^ Ha
Convergence tolerance	Self-consistent field density convergence (SCF)	2 × 10^−5^
	Binding energy tolerance	2 × 10^−5^ Ha
	Force tolerance	4 × 10^−3^ Ha Å^−1^
D2 correction^42^	Exchange-correlation dependent factor s6	1.0
	Damping coefficient *d*	20.0

## 3 Results and discussion

### 3.1 Adsorption of alkanes, cycloalkanes and aromatics on the Ni–Mo–S active site

The adsorption of saturated hydrocarbons on the Ni–Mo-edge is a prerequisite for dehydrogenation. To a certain extent, the interactions between the HOMO of hydrocarbons and the unoccupied molecular orbitals of the active sites contribute to the adsorption morphology and energy. In this study, the initial setting of the adsorption morphology was based on full contact with the molecular orbitals, whereas the optimized morphology was defined according to the minimum energy principle after atomic coordinate adjustments. The HOMO and adsorption parameters of alkanes, cycloalkanes and aromatics on the Ni–Mo edge are shown in Table 2. According to the calculation results, the HOMOs of the C-10 and C-14 alkanes were evenly distributed along each C–C single bond. The eigenvalues were between 6.80–7.00 eV. The HOMO distribution in cycloalkanes was similar to that in alkanes, whereas the eigenvalues were approximately 0.5 eV higher. In other words, the HOMOs of saturated hydrocarbons were blocked by the single C–C bond. Therefore, the adsorption of saturated hydrocarbons on the metal active sites would rely on the van der Waals force. Up to a certain carbon number, the adsorption intensity of the saturated hydrocarbons increased with the contact area on the active sites, and the adsorption energy increased with the molecular weight. The adsorption energy of the C-14 saturated hydrocarbons was approximately 20–30 kJ mol^−1^ larger than that of C-10.

**Table 2:** Adsorption of alkanes, cycloalkanes and aromatics on the Ni–Mo-edge

Hydrocarbons	HOMO eigen value/eV	Morphology	Binding energy kJ mol^−1^	Gibbs free energy kJ mol^−1^
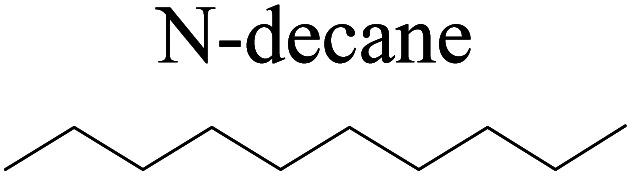	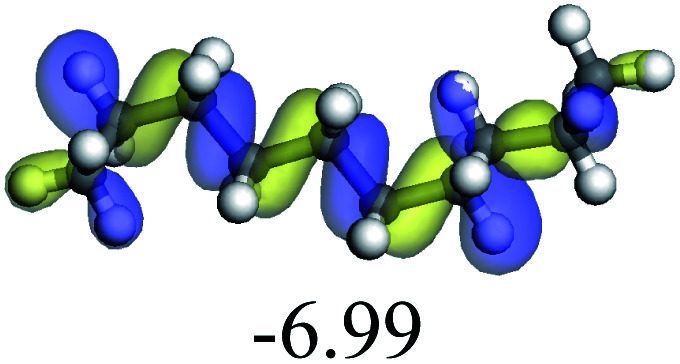	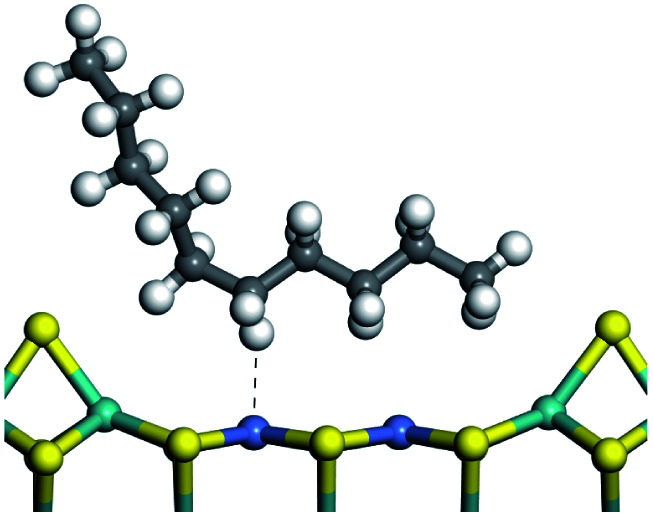	−54.56	−10.69
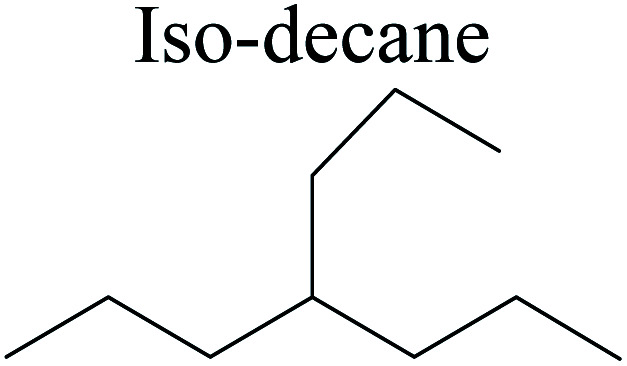	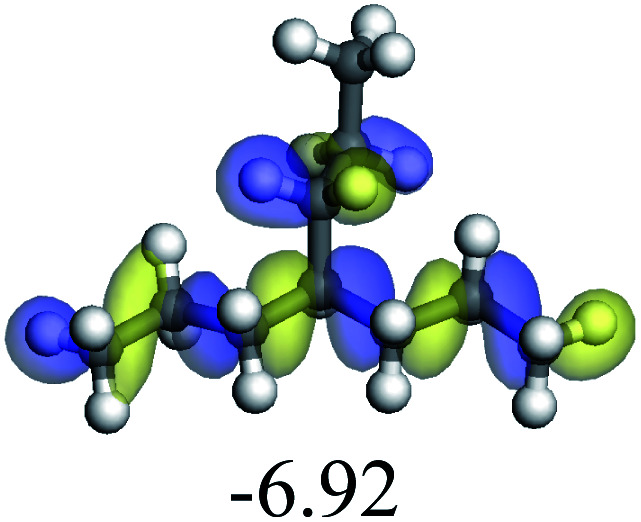	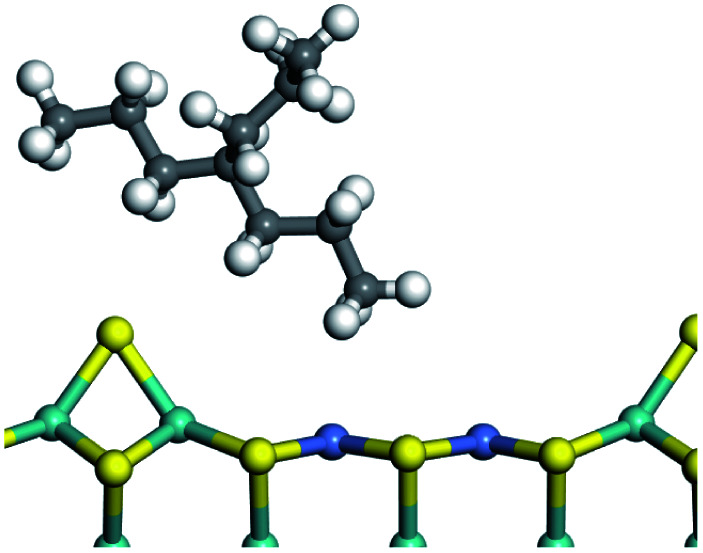	−51.93	−3.44
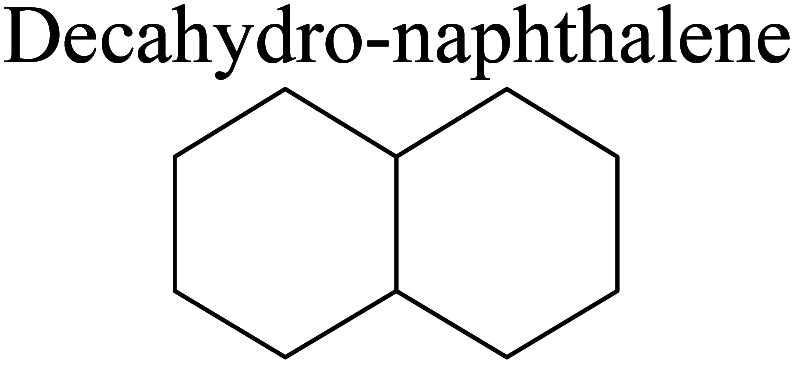	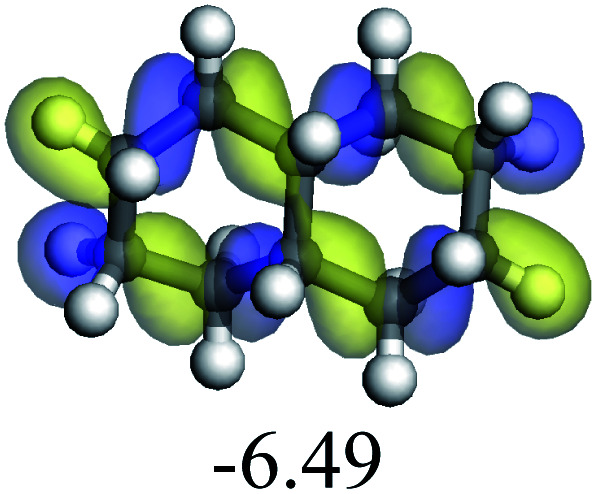	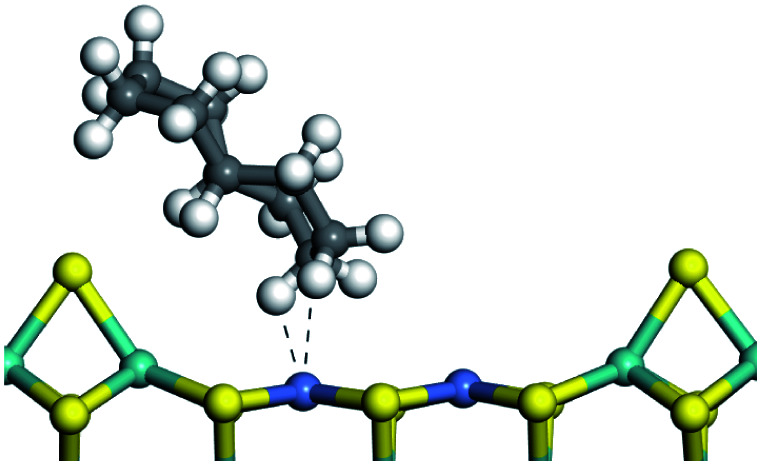	−57.81	−12.60
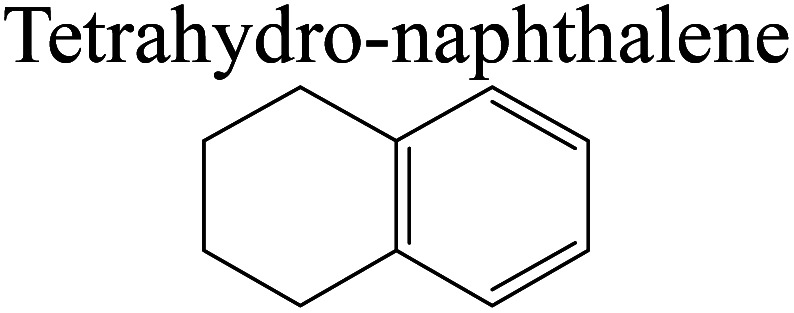	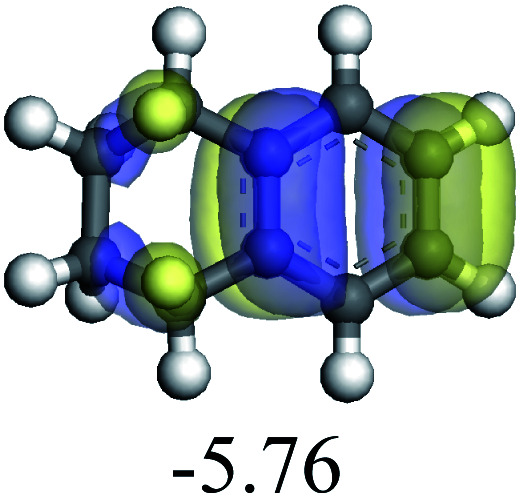	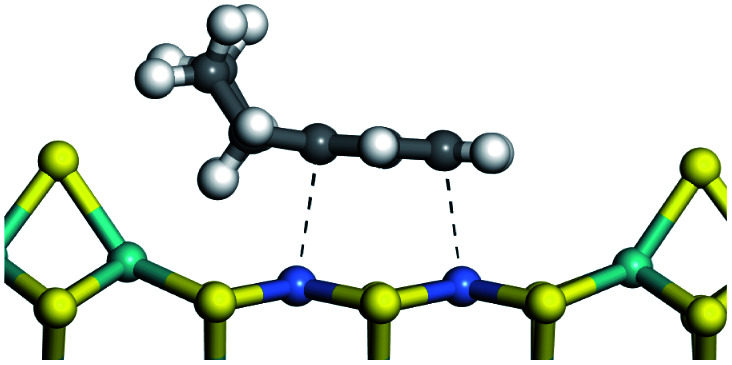	−101.93	−43.95
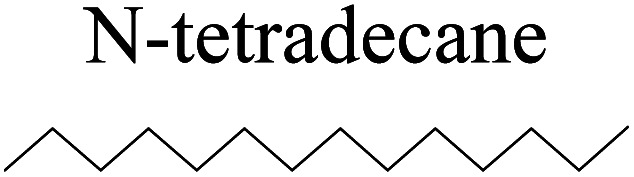	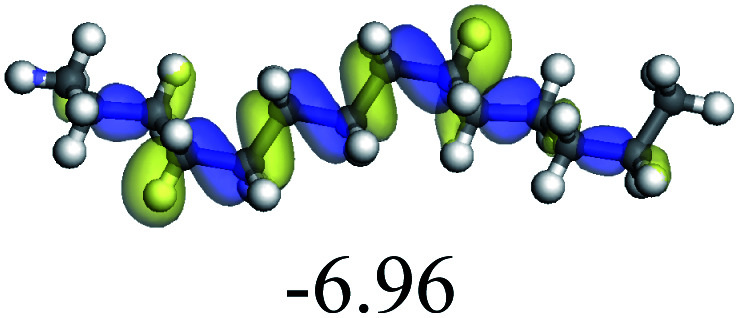	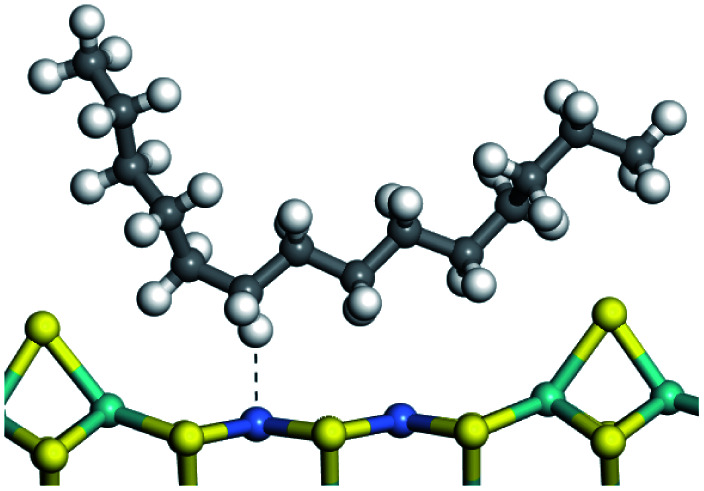	−81.09	−36.93
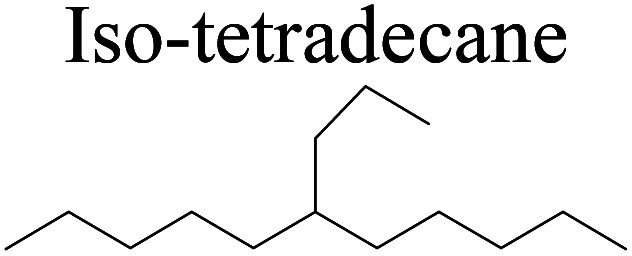	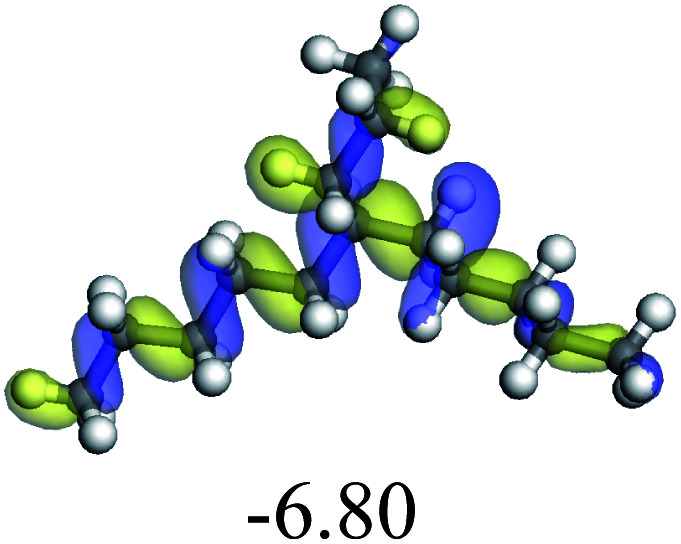	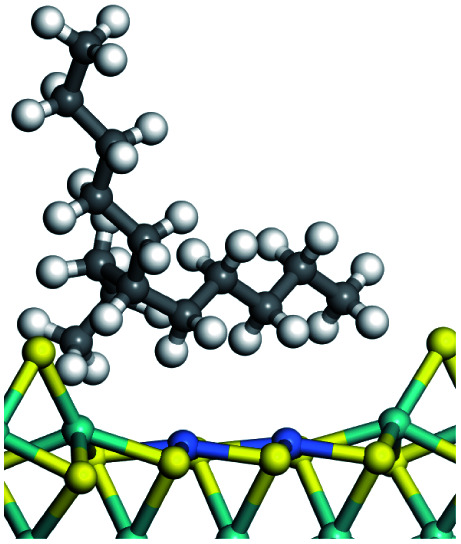	−76.27	−33.20
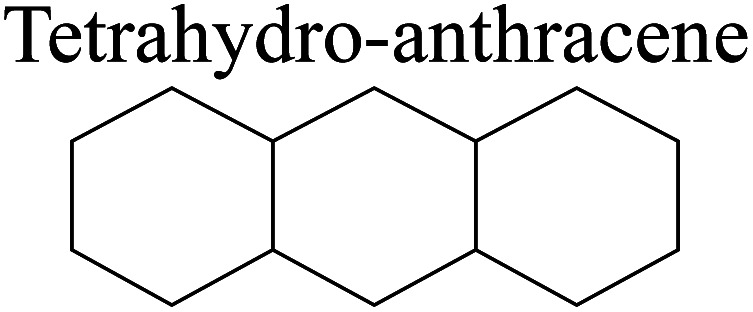	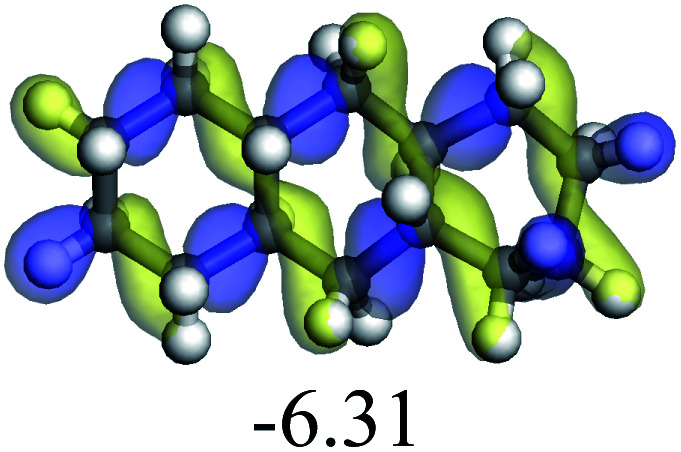	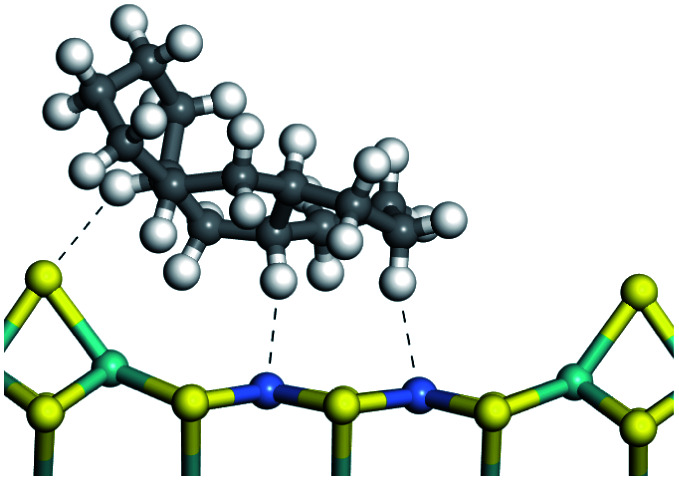	−89.20	−45.36
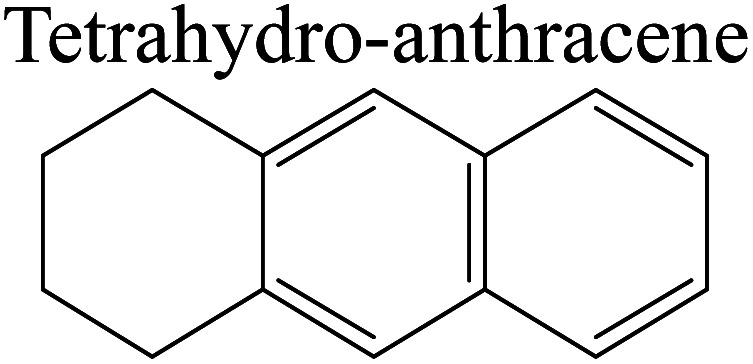	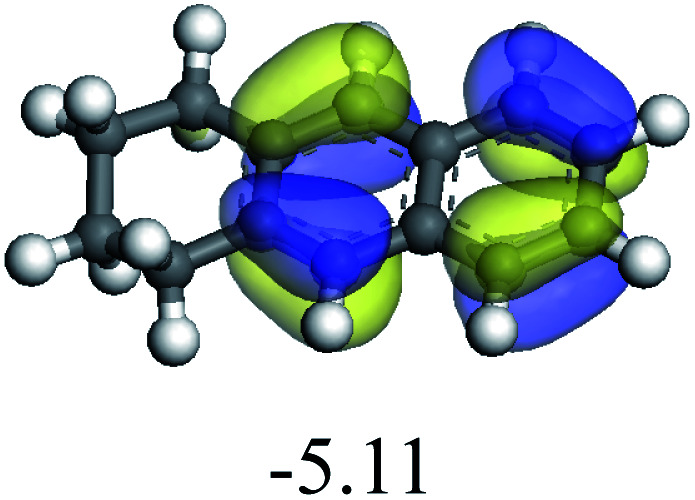	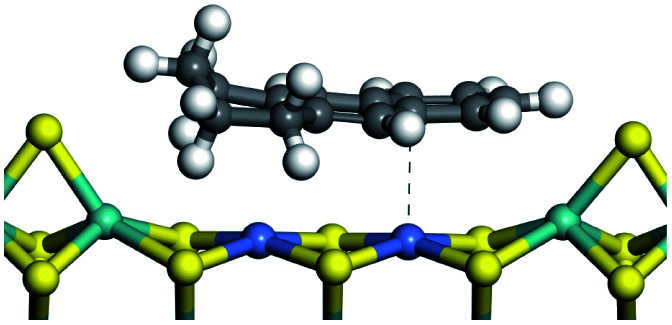	−126.91	−86.57
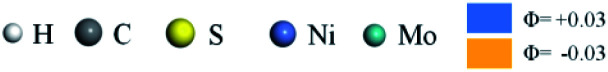				

On the other hand, the HOMOs of the aromatics were distributed on both sides of the aromatic rings where the conjugated π-electrons existed. These HOMOs had good contact with the exposed Ni atoms on the Ni–Mo-edge active sites. The adsorption energy of aromatics was 40–50 kJ mol^−1^ higher than those of alkanes and cycloalkanes because some aromatic carbon atoms could interact with the Ni atom. The adsorption energy of tetrahydroanthracene was as high as −126.91 kJ mol^−1^, and the Gibbs free energy was −73.57 kJ mol^−1^. According to the calculation results, it could be predicted that the aromatics had an advantage in competitive adsorption compared with saturated hydrocarbons. The normal alkanes and cycloalkanes take the second place, and the iso-alkanes are at a disadvantageous position.

### 3.2 Dehydrogenation of alkanes, cycloalkanes and aromatics on the Ni–Mo–S active site

The saturated hydrocarbons need to be converted to olefins on the metal active sites for the cracking process to continue on the zeolite acid centers. The dehydrogenation of hydrocarbons is a complex process, which can be decomposed into three elementary reactions: the generation of radical carbon, the generation of C=C double bonds, and the generation of hydrogen molecules. The dehydrogenation data of a C-10 normal alkane, an iso-alkane and a cycloalkane are listed in Table 3. According to the calculation results, the generation of radical carbon is apparently a very strong endothermic process (for any reaction, the reaction energy equals *E*(products) − *E*(reactants), and the reaction barrier equals *E*(TS) − *E*(reactants). From the trend of the energy change, one can calculate the reaction energy and reaction barrier.) with the reaction energy of +250.09 plus −70.82, which equals 179.27 kJ mol^−1^. The activation energy was up to 250.09 kJ mol^−1^, indicating that this step was no doubt the rate-controlling step of the alkane dehydrogenation reaction and even the entire hydrocracking process. The intensive rise in energy is mainly attributed to the large energy difference between the breaking of the C–H bond and the generation of the –SH bond. The bond energy of C–H in alkanes is approximate 414 kJ mol^−1^,^43^ whereas the bond energy of –SH on the Mo-edge is expected to be 220–230 kJ mol^−1^.^19,30^ The following two steps, namely the generation of the C=C double bonds and hydrogen molecule, stabilize the system by merging the carbon and hydrogen radicals and forming a strong covalent bond. Therefore, these two steps are both exothermic with low activation energy, indicating quick conversion on the metal active sites. The dehydrogenation of iso-alkanes and cycloalkanes on the Ni–Mo–S active sites was quite similar to that of normal alkane. The generation of carbon radical was the evident rate-controlling step with high activation energy and the following two steps were also exothermic with low activation energy (Table 4).

**Table 3:** Dehydrogenation of C-10 normal alkane, iso-alkane and cycloalkane

Process	Alkane	TS-1	Radical	TS-2	C=C bonds	TS-3	H_2_
Morphology	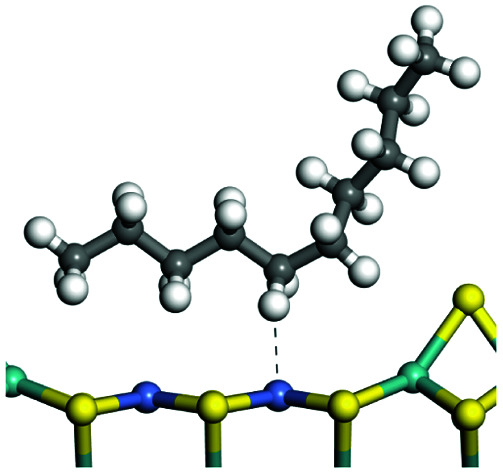	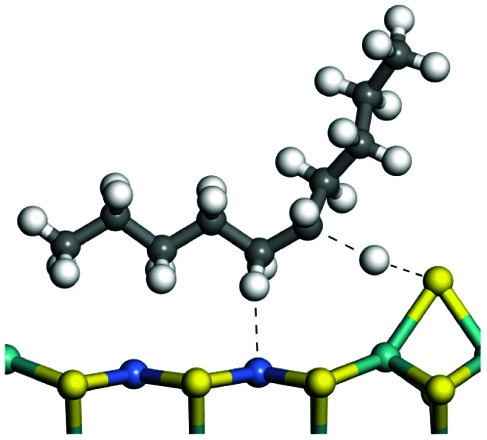	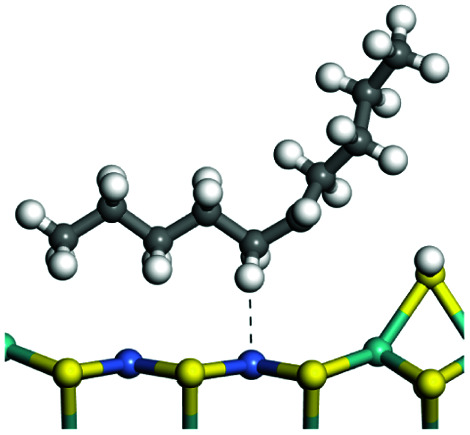	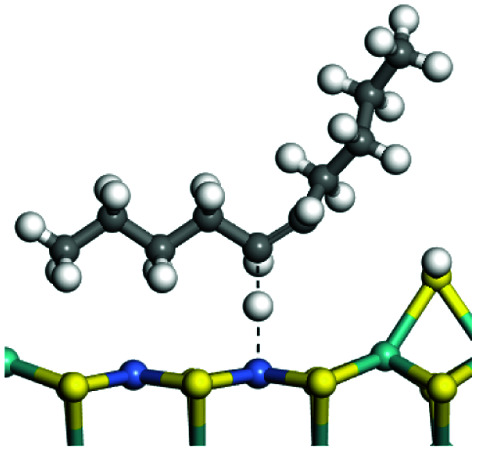	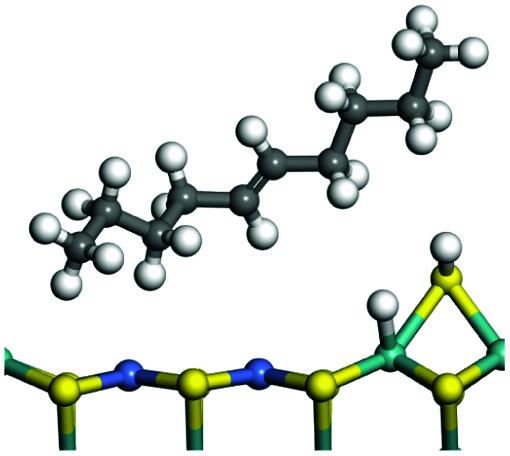	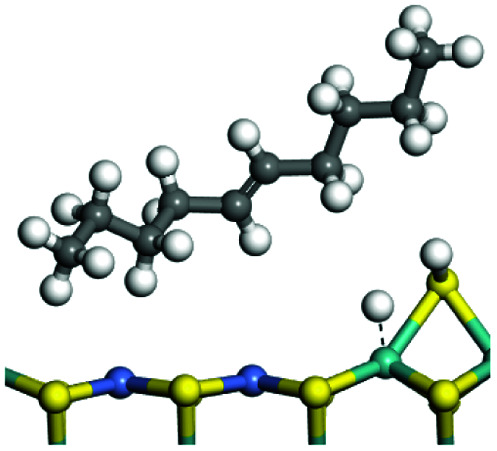	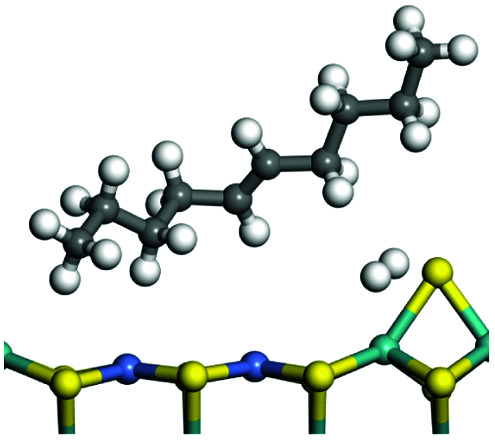
Bonding energy change kJ mol^−1^	N-decane	+255.09	−65.82	+86.44	−117.06	+100.72	−122.28
Morphology	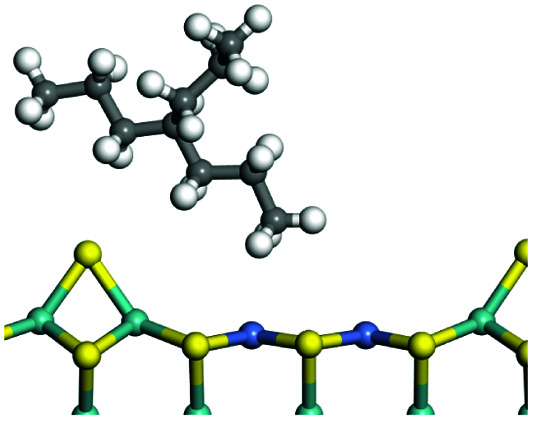	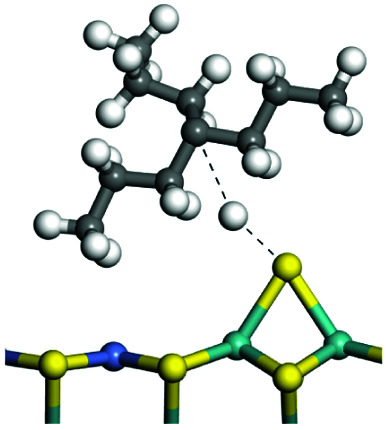	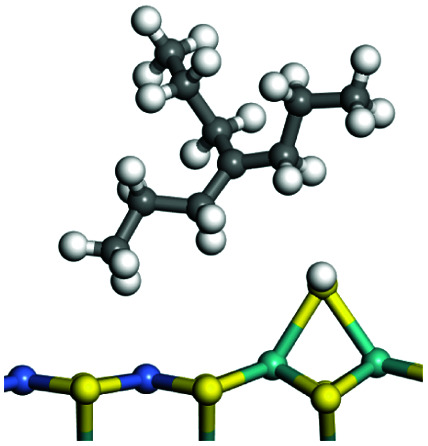	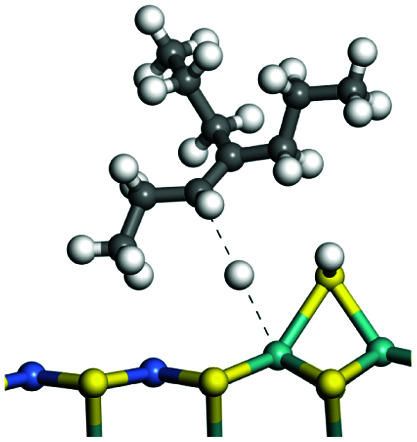	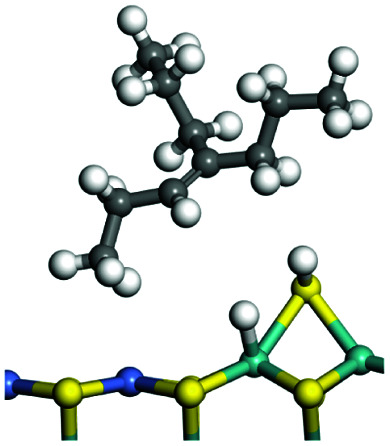	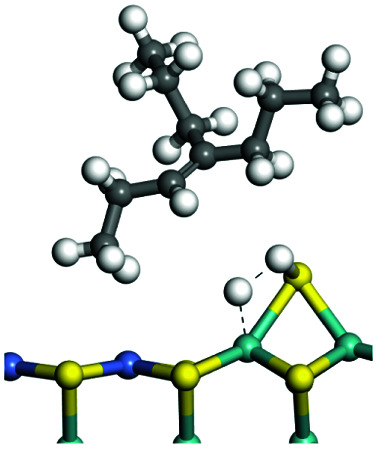	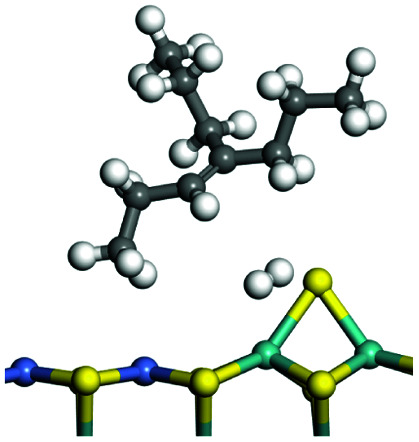
Bonding energy change kJ mol^−1^	Iso-decane	+242.79	−61.71	+116.02	−169.75	+117.00	−140.34
Morphology	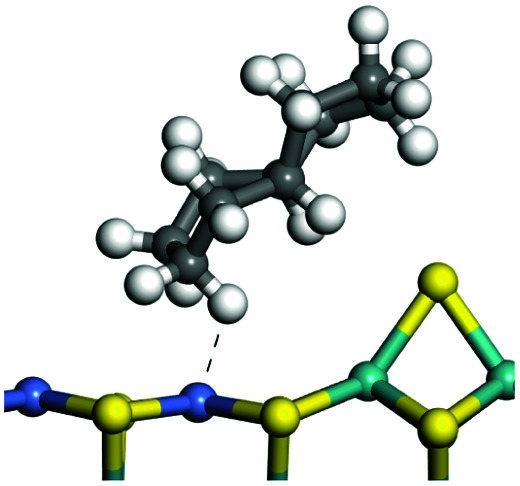	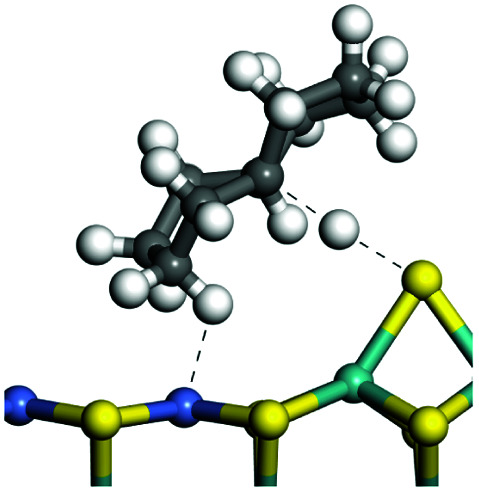	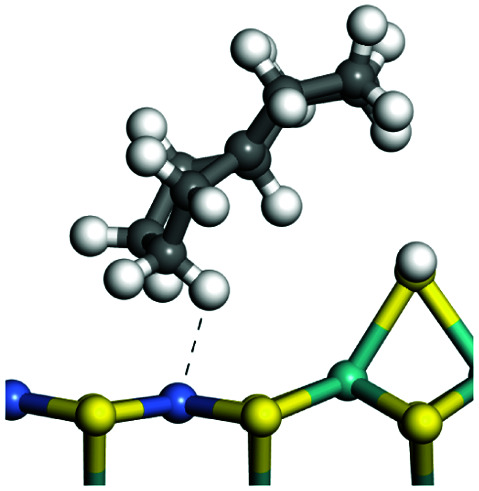	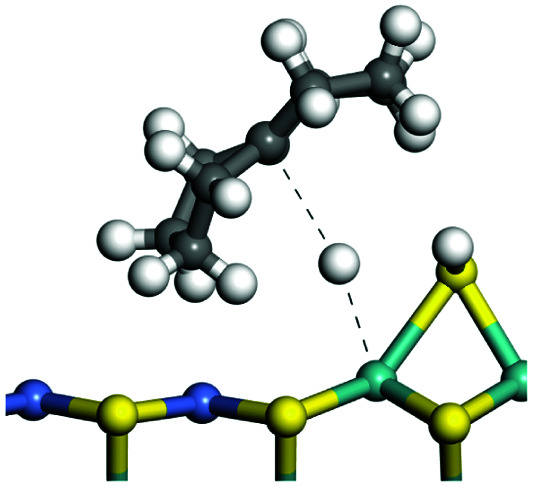	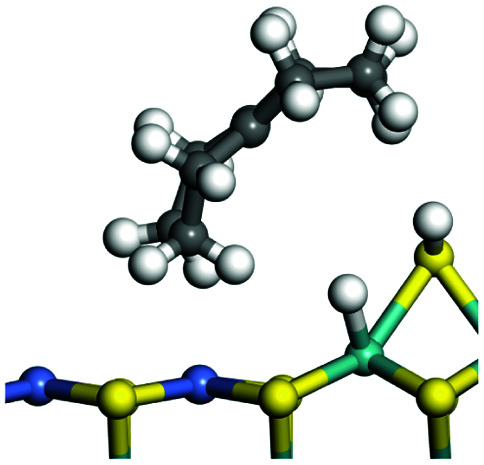	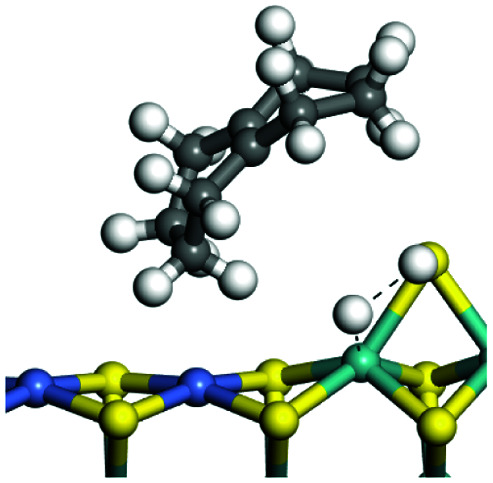	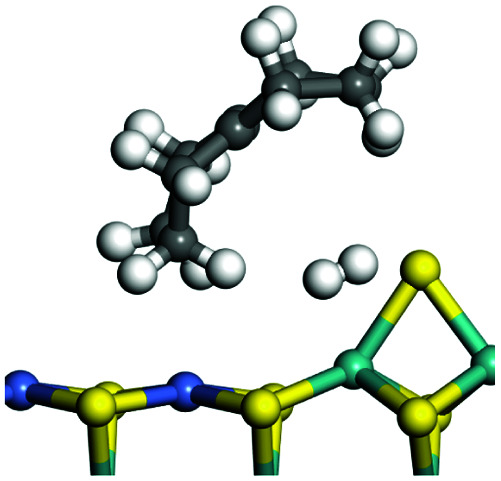
Bonding energy change kJ mol^−1^	Decahydro-naphthalene	+245.67	−72.07	+132.41	−166.55	+113.96	−139.20

**Table 4:** Dehydrogenation of C-14 normal alkane, iso-alkane and cycloalkane

Process	Alkane	TS-1	Radical	TS-2	C=C bonds	TS-3	H_2_
Morphology	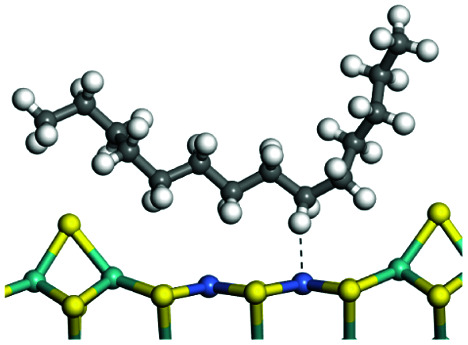	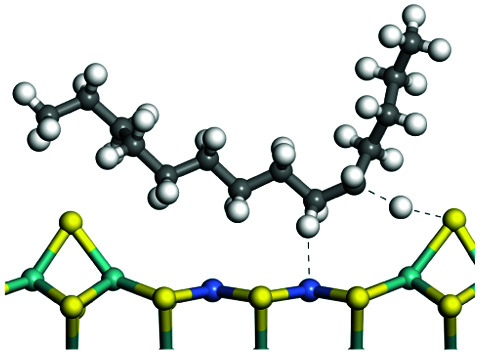	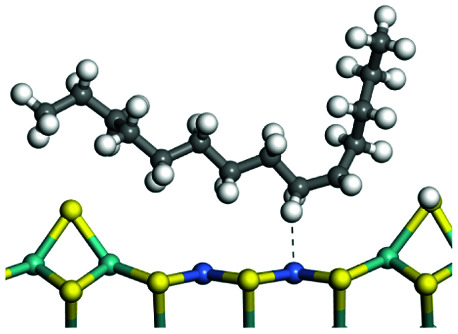	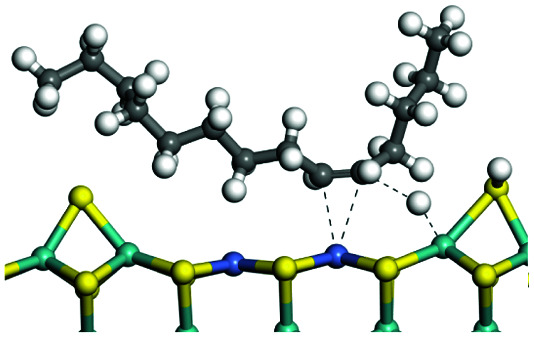	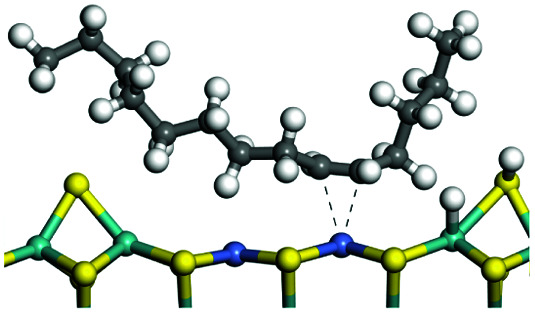	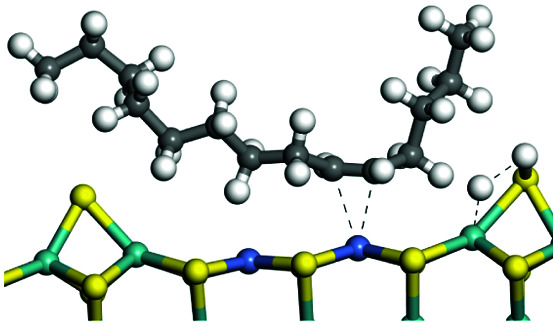	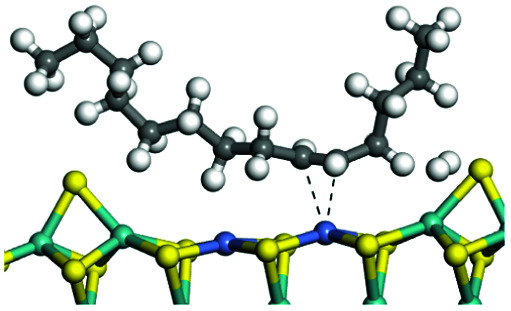
Bonding energy change kJ mol^−1^	N-tetradecane	+261.56	−72.91	+95.68	−132.50	+103.68	−140.05
Morphology	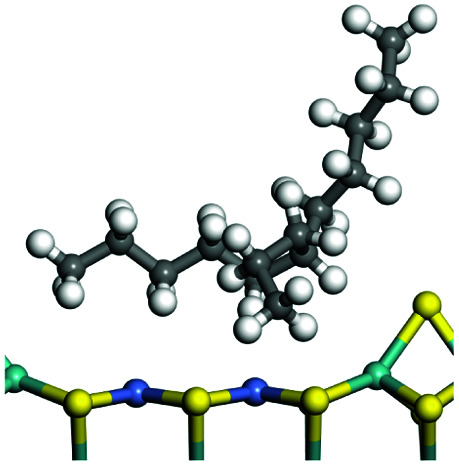	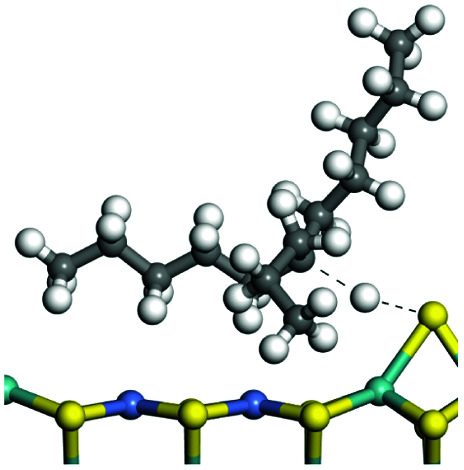	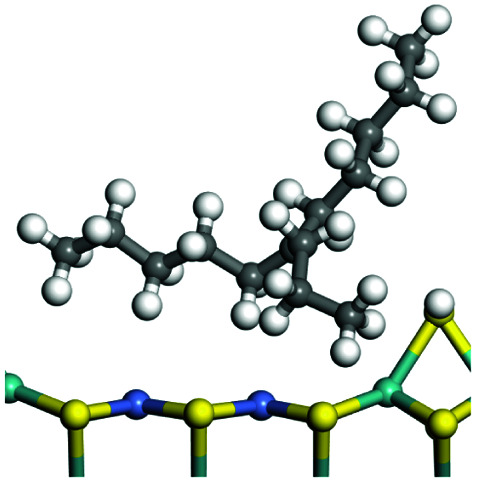	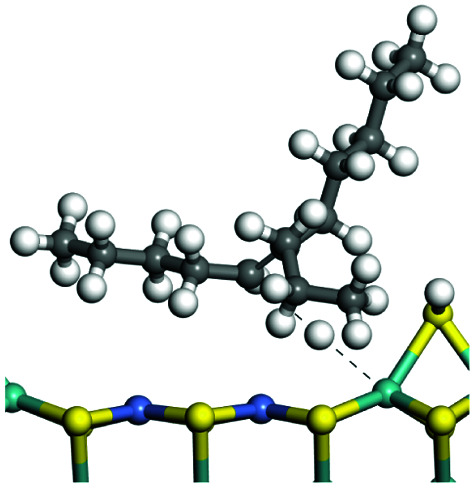	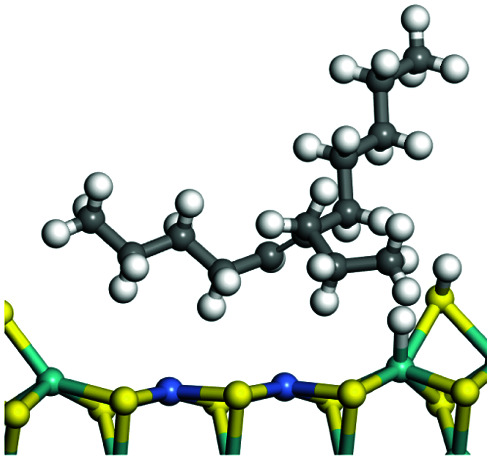	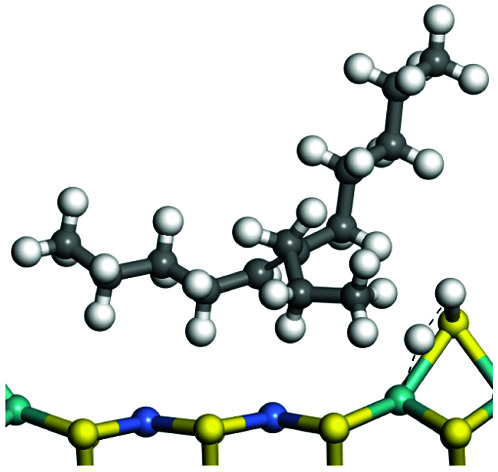	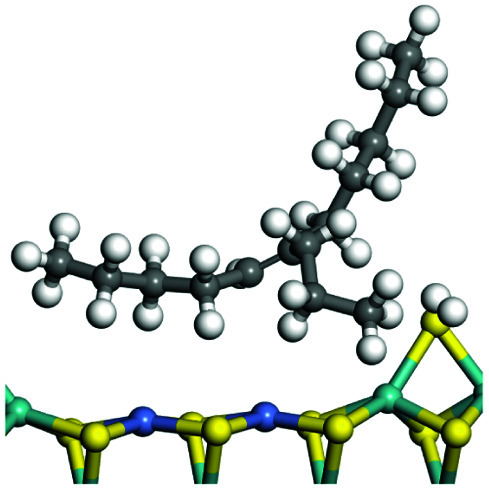
Bonding energy change kJ mol^−1^	Iso-tetradecane	+241.21	−65.11	+84.27	−134.51	+112.46	−137.22
Morphology	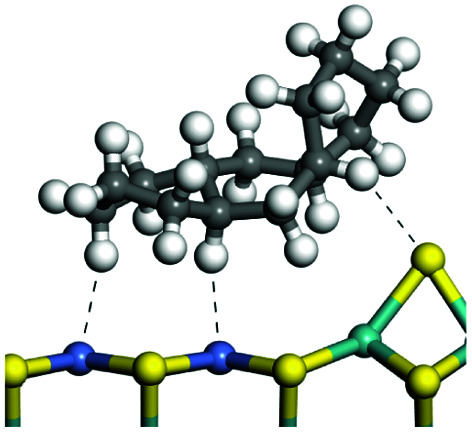	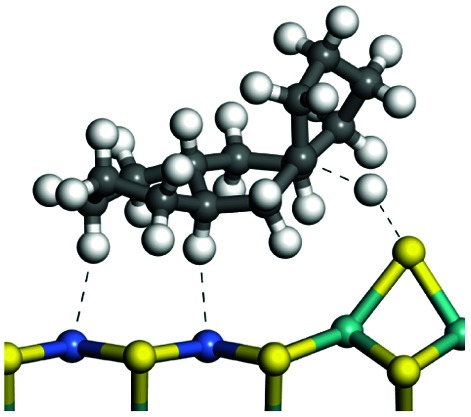	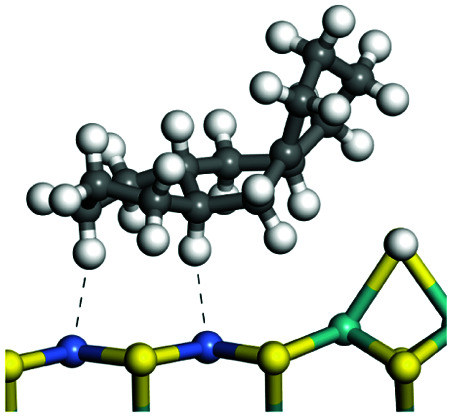	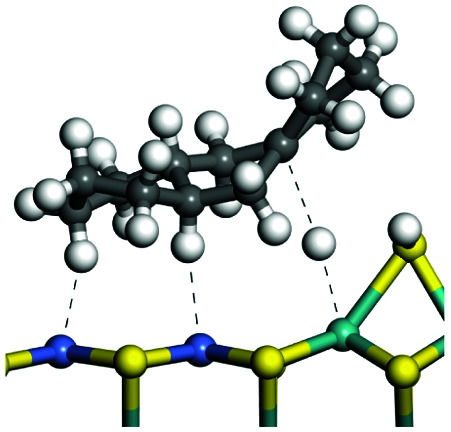	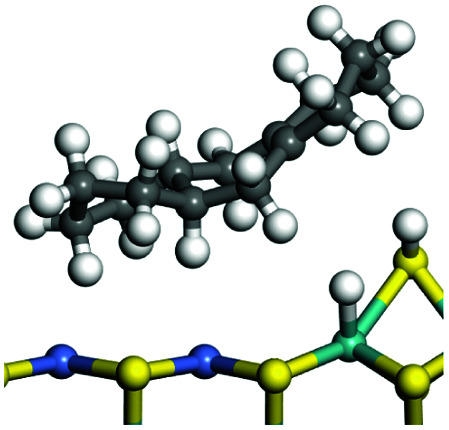	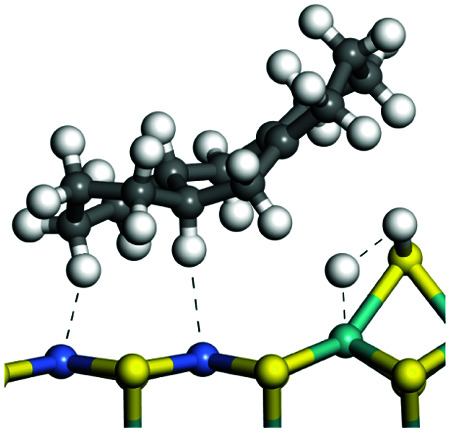	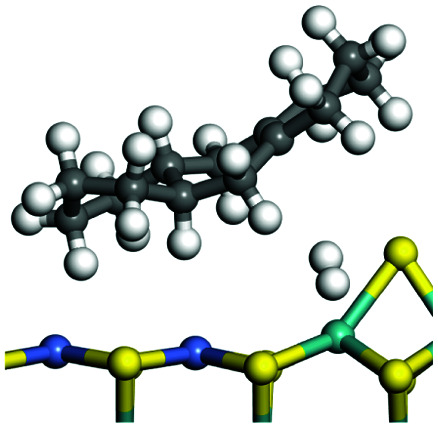
Bonding energy change kJ mol^−1^	Decahydr-anthracene	+244.53	−70.62	+126.43	−165.88	+99.65	−115.90

In addition, the total energy change during the dehydrogenation of saturated hydrocarbons was 120–130 kJ mol^−1^. Considering the strong competitive adsorption of the aromatics and high hydrogen pressure in the reaction system, the equilibrium conversion of hydrocarbon dehydrogenation is expected to be low.

### 3.3 Adsorption of alkenes, cyclic alkenes and aromatics on FAU-zeolite

**Fig. 3:**
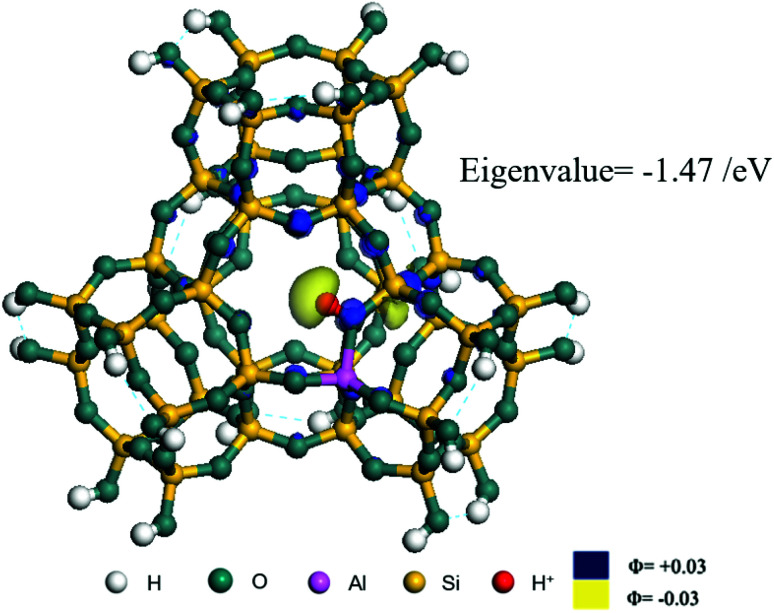
The LUMO morphology on FAU-Al.

The competitive adsorption of unsaturated hydrocarbons on zeolite can also affect the cracking sequence. The adsorption results of alkenes, cyclic alkenes and aromatics on Al-FAU are shown in Table 5. Similar to the metal active sites, the adsorption of hydrocarbons on the zeolite could be explained by the frontier molecular orbitals. The LUMO morphology of FAU-Al is shown in Fig. 3. The LUMO was located on the H^+^ near the Al atom and pointed to the super cage of FAU. The HOMOs of alkenes and aromatics mainly consist of π-electron pairs. The HOMO eigenvalues increase with the branching of unsaturated carbons and the conjugate area on the aromatic rings. The adsorption energy of 5-decene, 4-propyl-3-heptene and octahydronaphthalene were also enhanced with the HOMO eigenvalues. Because of the better accessibility of the HOMO on the aromatic ring, the adsorption energy of tetrahydronaphthalene was larger than those of the saturated hydrocarbons, and even the eigenvalue was lower. On the other hand, the difference in adsorption caused by molecular weight was even larger. The additional four carbon atoms contributed an extra 25–35 kJ mol^−1^ of adsorption energy, which could be attributed to the dispersion force. Without considering the limitation of the pore structure, the larger molecule with long substituent groups stretches long the rough surface.

**Table 5:** Adsorption of unsaturated hydrocarbons on FAU-Al

Hydrocarbons	HOMO eigenvalue/eV	Morphology	Binding energy kJ mol^−1^	Gibbs free kJ mol^−1^
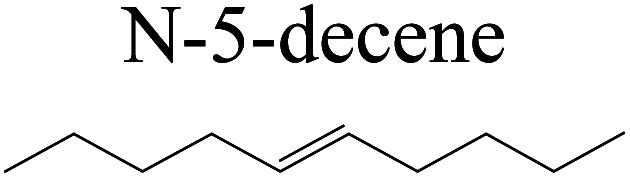	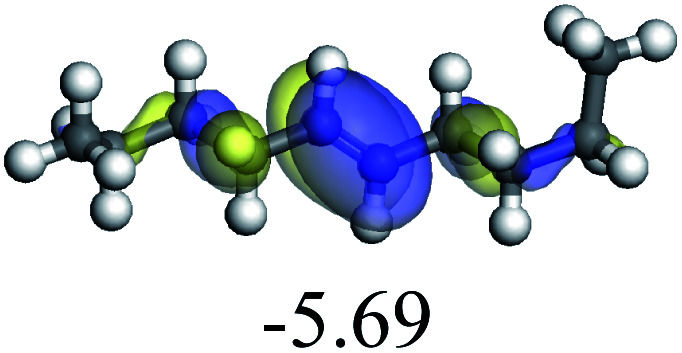	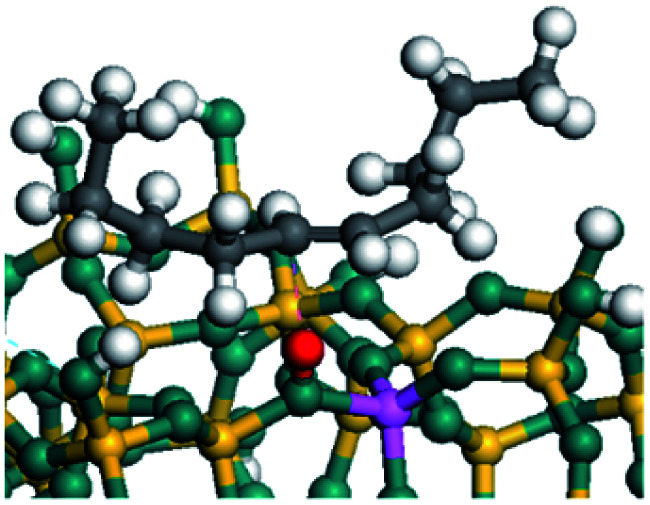	−58.28	−10.96
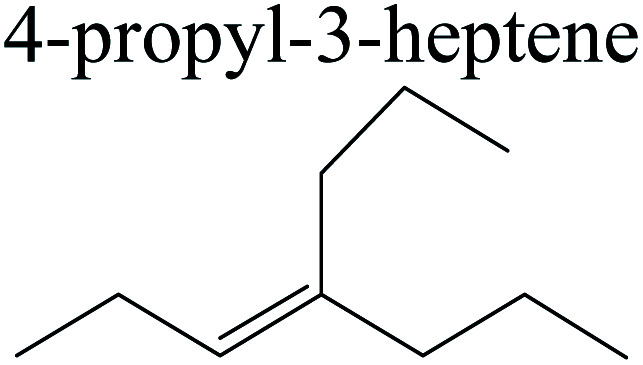	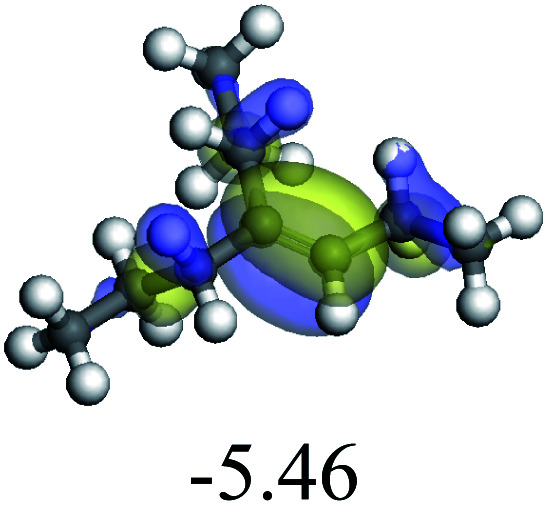	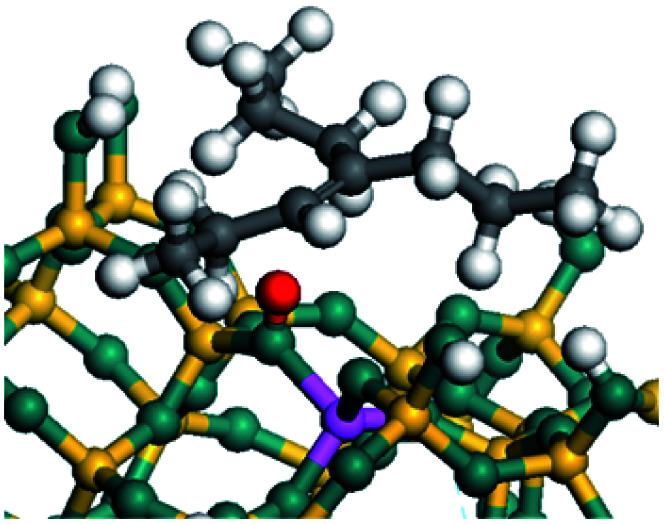	−60.55	−15.33
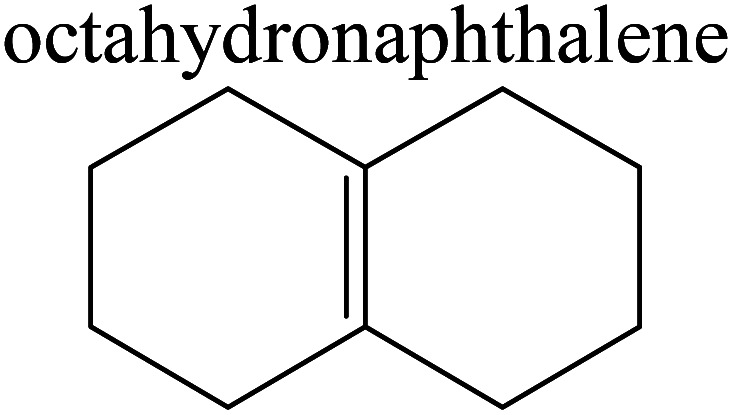	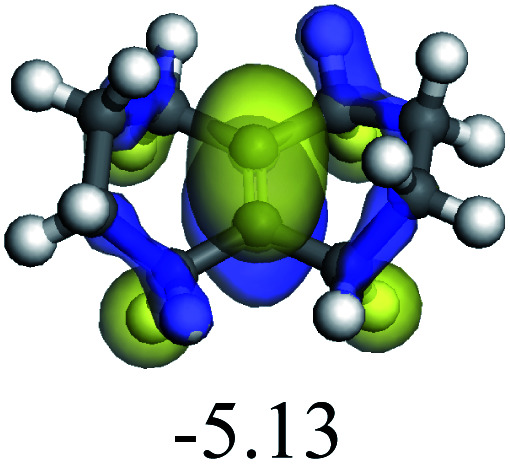	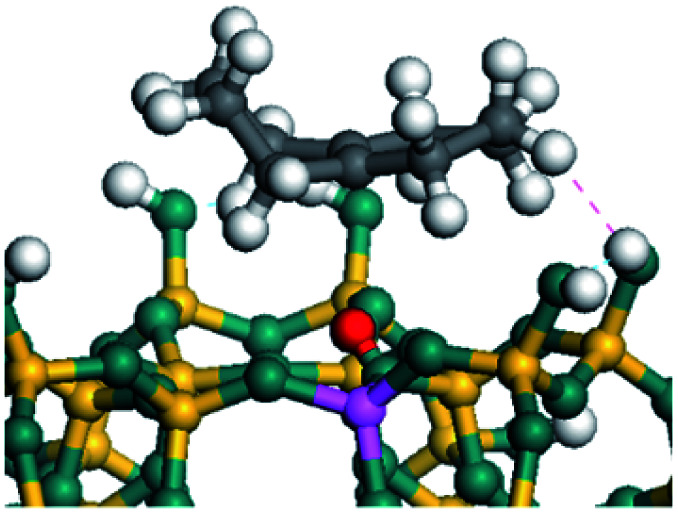	−64.07	−16.60
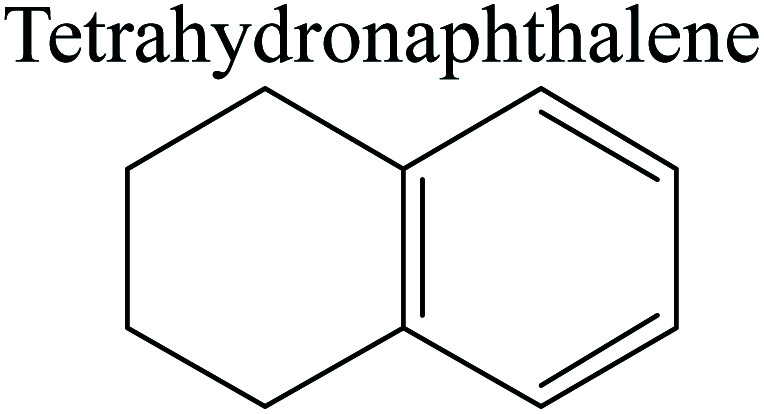	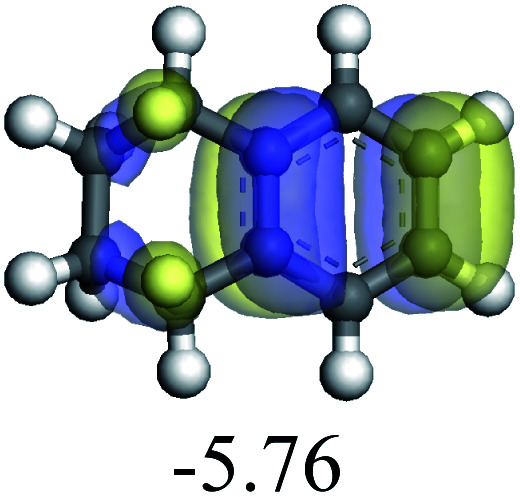	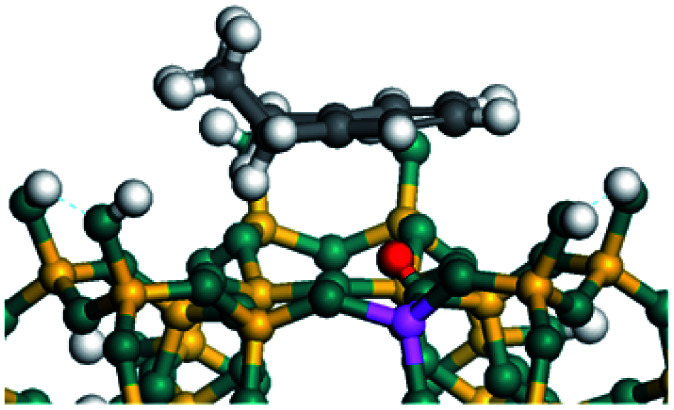	−70.46	−22.53
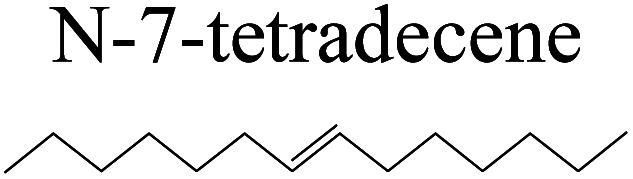	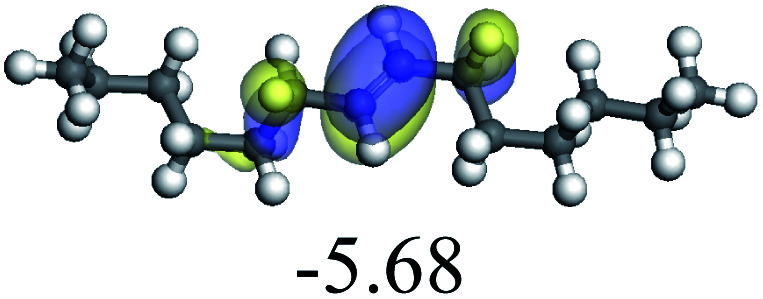	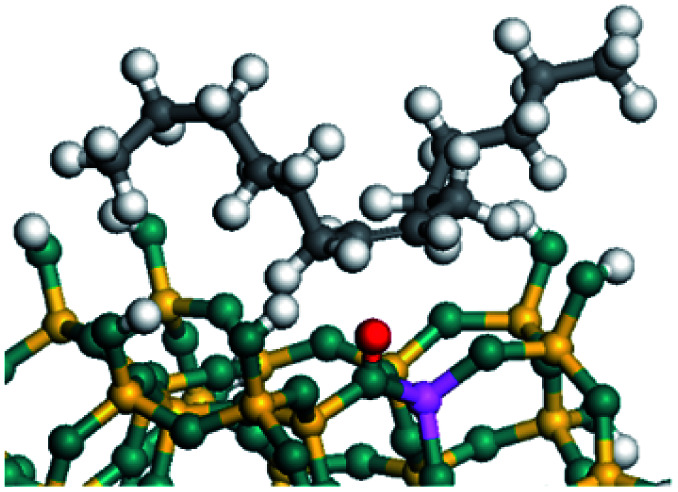	−83.26	−39.00
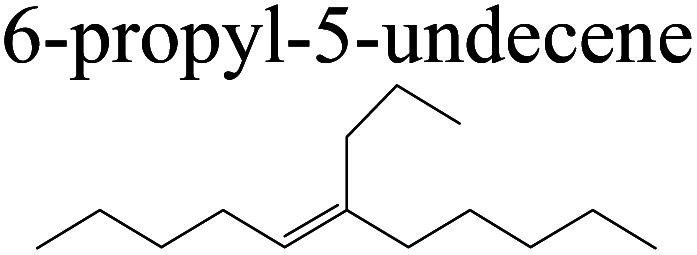	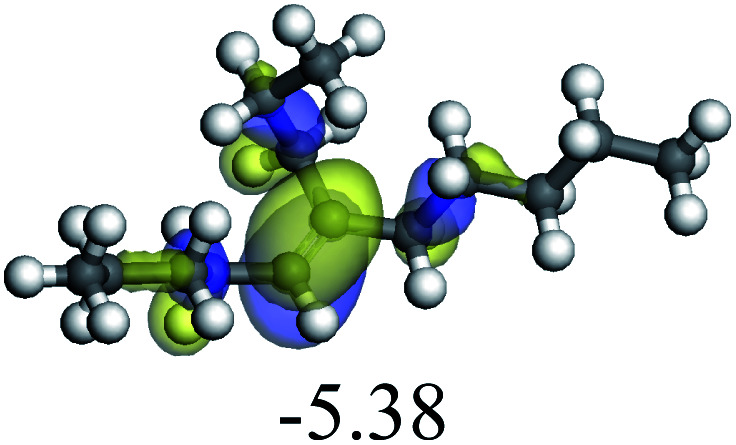	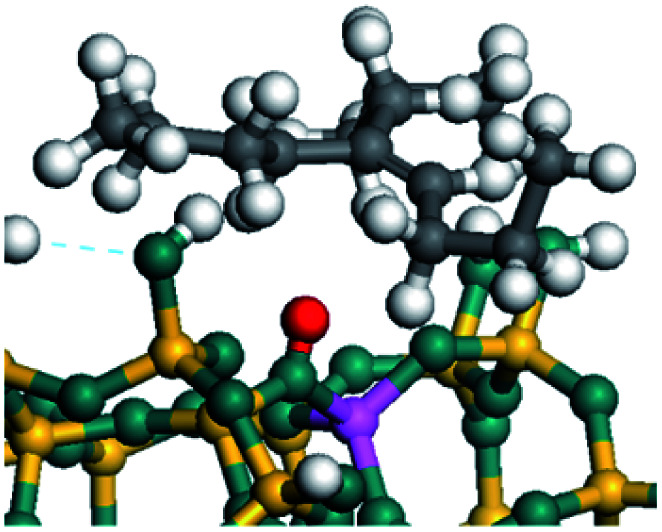	−92.39	−46.29
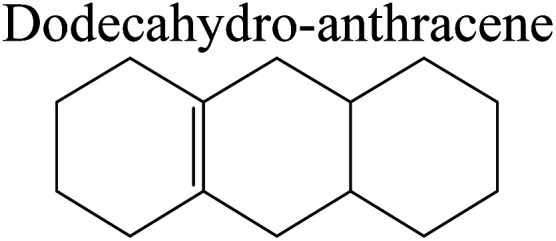	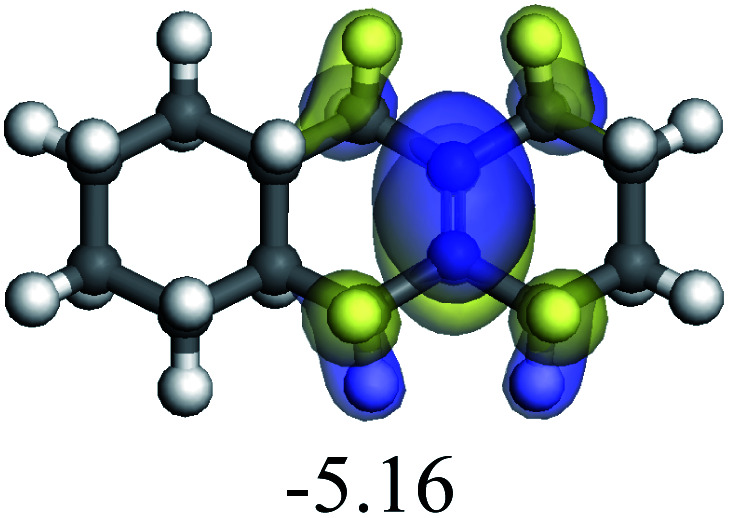	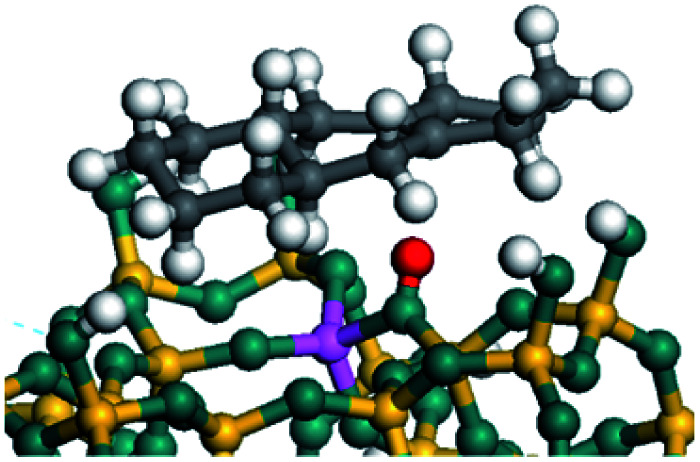	−97.69	−53.14
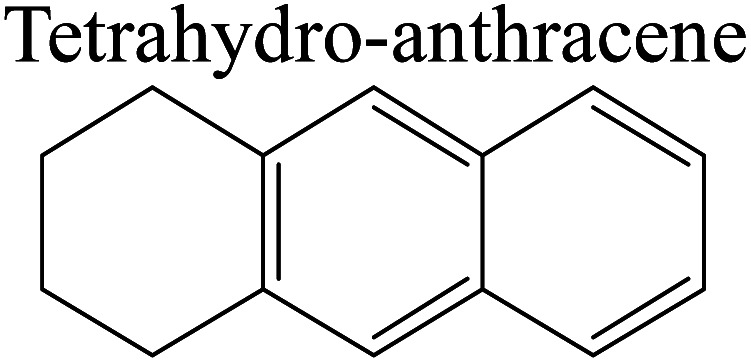	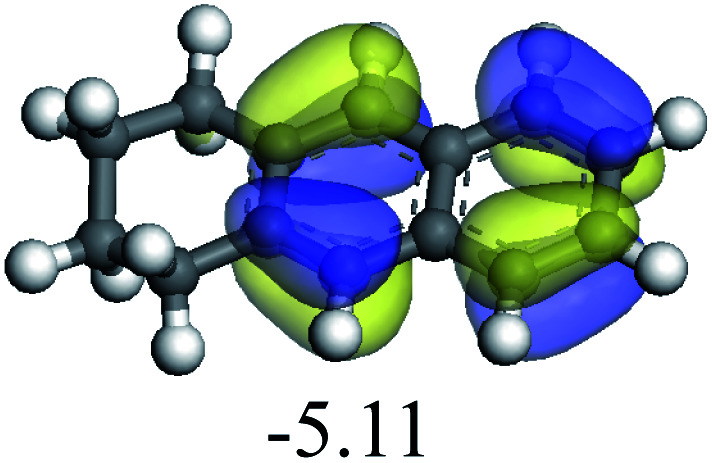	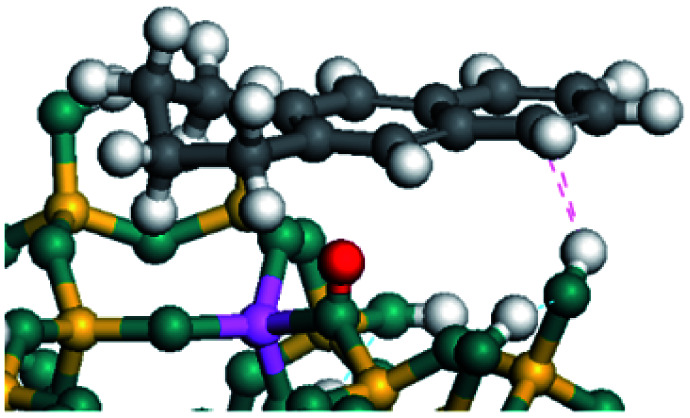	−102.08	−60.22

### 3.4 Protonation of alkenes, cyclic alkenes and aromatics on the zeolite

In this study, the Mulliken charge was used to evaluate the charge redistribution and transfer between the hydrocarbons and the zeolite. It should be noted that the Mulliken charge is just a horizontal comparison to estimate the charge differences brought about by the hydrocarbon structures, and the results are entirely suitable for predicting the other physicochemical properties of the catalytic system. Protonation is the step in which the positive hydrogen, which takes +0.391 Mulliken charge, on Al–O–H transfers to the unsaturated C=C group, forming a neutral sp^3^ carbon and a positive sp^2^ carbon. The stability of the carbonium ion is related to the electrostatic distribution of the hydrocarbons. The protonation and charge distribution in alkenes, cyclic alkenes and aromatics on FAU-Al are shown in Table 6. On saturated hydrocarbons, the Mulliken charge of the carbonium ranged from +0.24 to +0.28, and the charge of the newly formed sp^3^ carbon (denoted as α-C) ranged from −0.23 to −0.28. The charge difference between C^+^ and α-C was 0.5–0.6*e*. The protonation of alkenes and cyclic alkenes was a moderate endothermic process with the reaction energy ranging between 10–30 kJ mol^−1^. In comparison, on tetrahydronaphthalene and tetrahydroanthracene, the carbonium only took approximately 0.15 positive charge, whereas the charge difference decreased to approximately 0.4*e*. With the breaking of the conjugated electrons, the protonation of tetrahydronaphthalene and tetrahydroanthracene was an obvious endothermic process with the reaction energy in the range of 50–70 kJ mol^−1^, indicating that, on the zeolites acid center, the protonation of the aromatic ring was more difficult than that of alkenes.

**Table 6:** Protonation of unsaturated hydrocarbons on FAU-Al

Carbonium	Morphology	Mulliken charge/*e*			Protonation energy kJ mol^−1^
		C^+^	α-C	α-H	
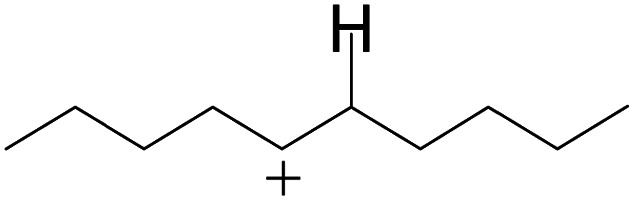	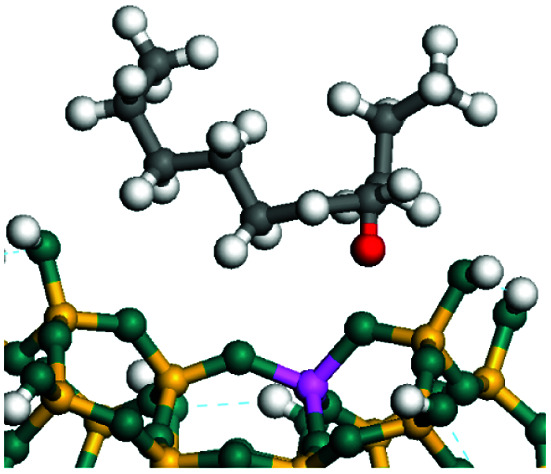	+0.242	−0.261	+0.327	+23.04
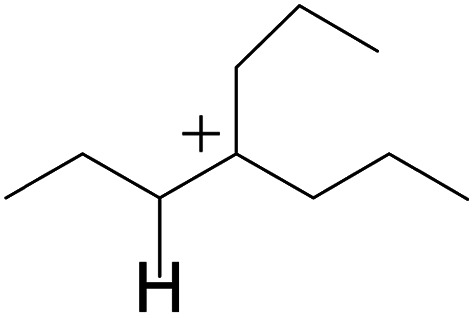	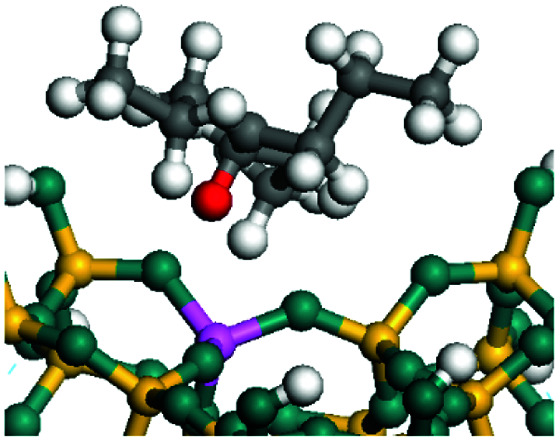	+0.250	−0.273	+0.319	+18.84
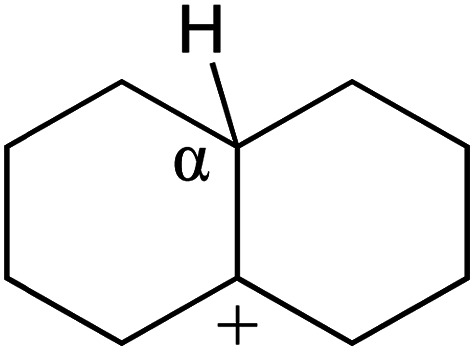	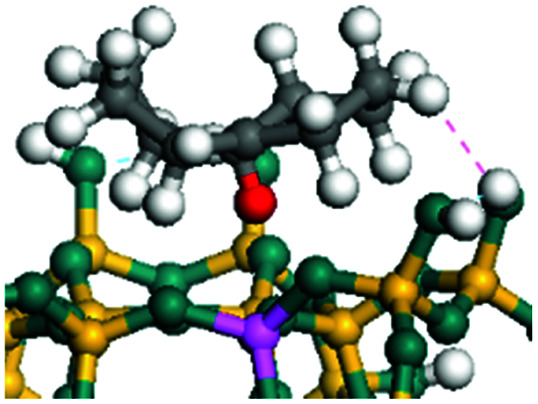	+0.274	−0.233	+0.312	+21.66
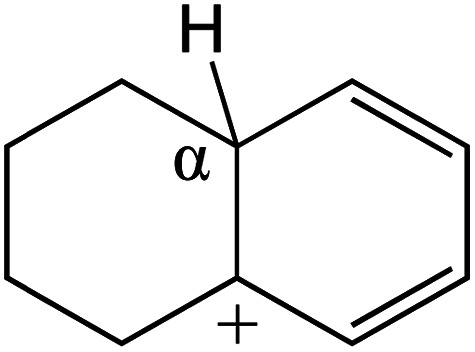	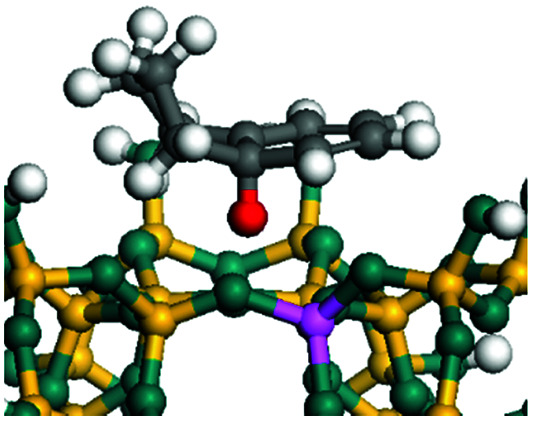	+0.172	−0.246	+0.329	+66.72
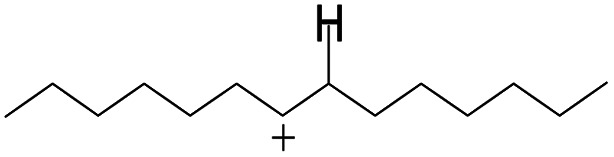	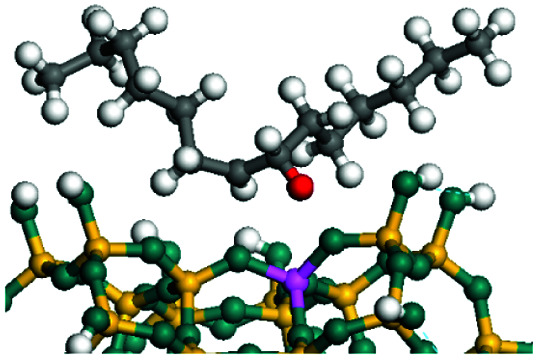	+0.245	−0.262	+0.333	+21.68
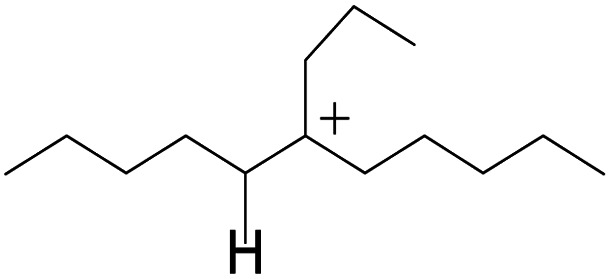	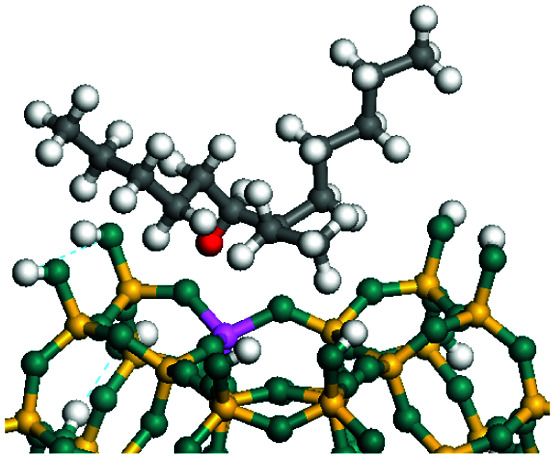	+0.241	−0.249	+0.326	+19.59
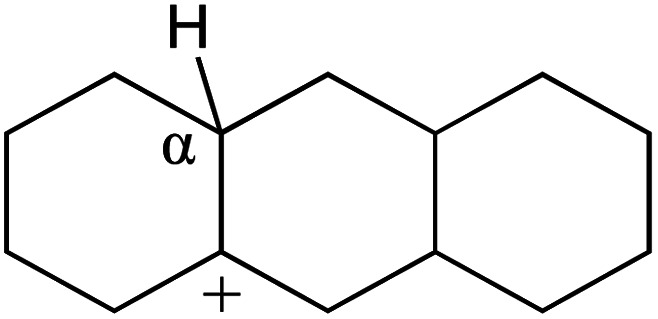	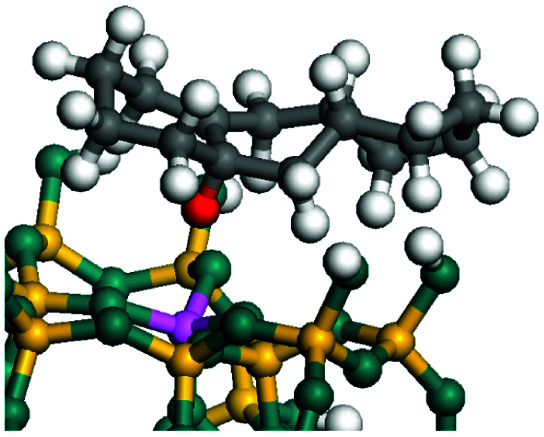	+0.272	−0.232	+0.298	+29.45
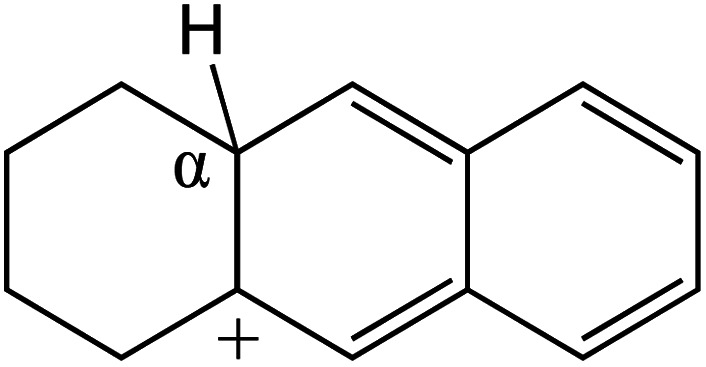	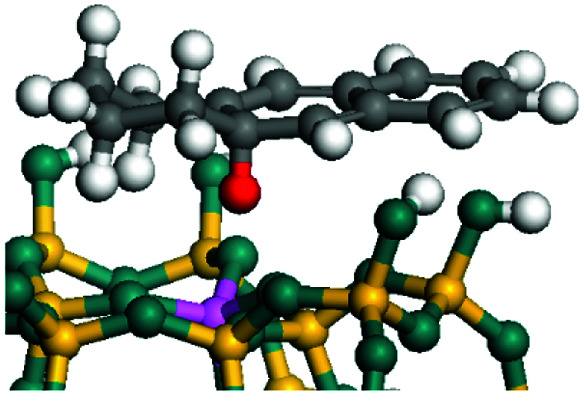	+0.140	−0.248	+0.303	+67.86

### 3.5 Cracking of alkenes, cyclic alkenes and aromatics on the zeolite

The cracking step of the hydrocarbons happens next, with the protonation of the C=C bond. In this step, the protonated alkanes divide into an olefin and a shorter protonated alkane. The breaking point is the β-position of C^+^, and the positive charge is transferred to the other side of the breaking point.^32,44^ The cracking steps of the C-10 and C-14 alkanes on FAU-Al are listed in Table 7. According to the calculation results, as the carbonium came to the end, it could strongly bond with the oxygen atom near the aluminum to lower the energy of the reactant. Further, the cracking of alkanes was a strong endothermic process with the reaction energy exceeding 60 kJ mol^−1^. The reaction energy of 3-propyl-heptane was even a bit higher because the stability of C-2 carbonium was lower than that of the long carbon chain. Furthermore, the activation energy during the cracking step was 170–200 kJ mol^−1^, which is approximately 110–120 kJ mol^−1^ higher than the reaction energy. The extra energy could be attributed to the separation of the newly formed carbonium (from the alkenes) that does not bond with the oxygen of FAU-Al in the transition state.

**Table 7:** Cracking of alkenes on FAU-Al

Cracking reaction	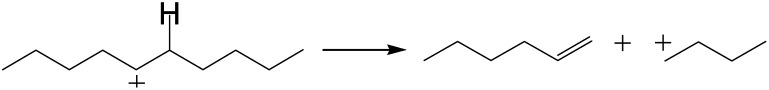			
Carbonium	Transition state	Reactant	Reaction energy kJ mol^−1^	Activation energy kJ mol^−1^
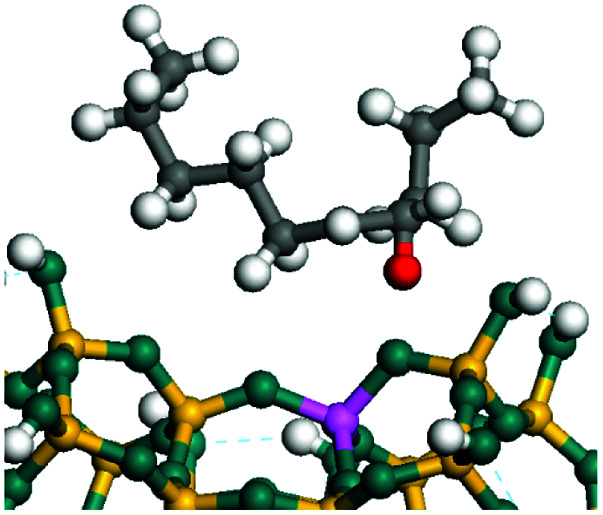	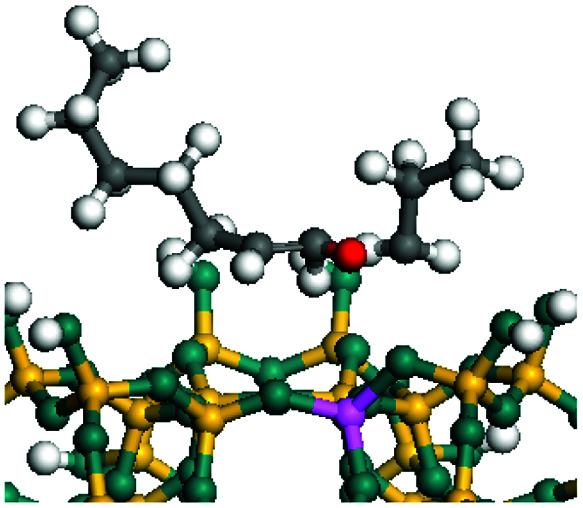	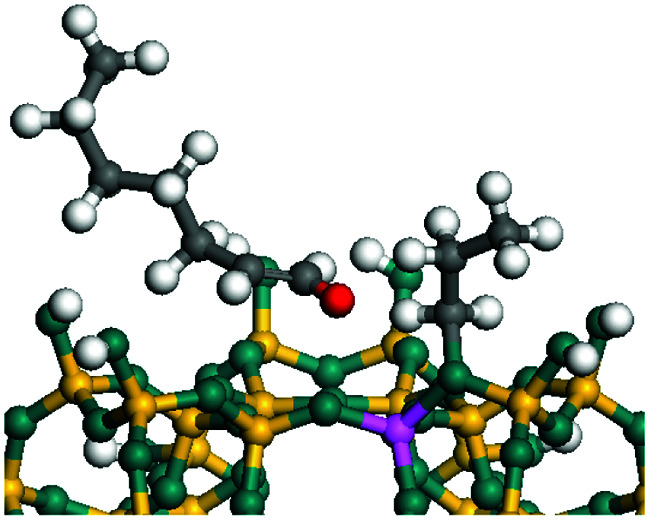	+69.37	+181.52
Cracking reaction	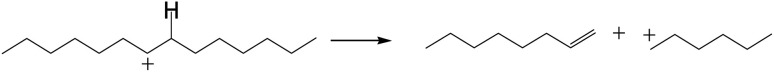			
Carbonium	Transition state	Reactant	Reaction energy kJ mol^−1^	Activation energy kJ mol^−1^
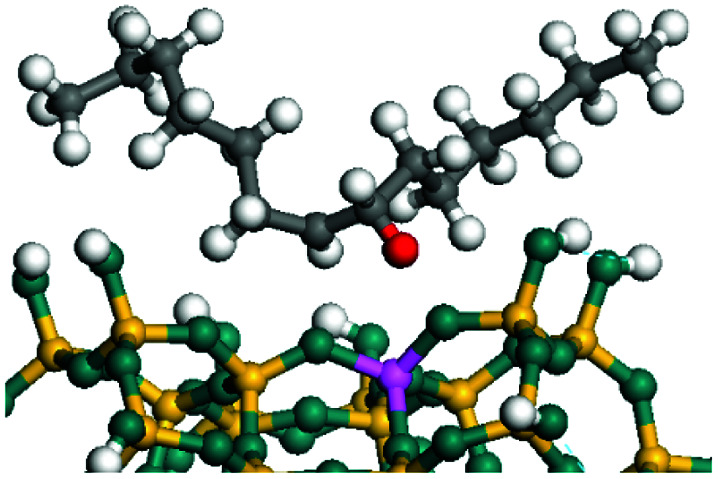	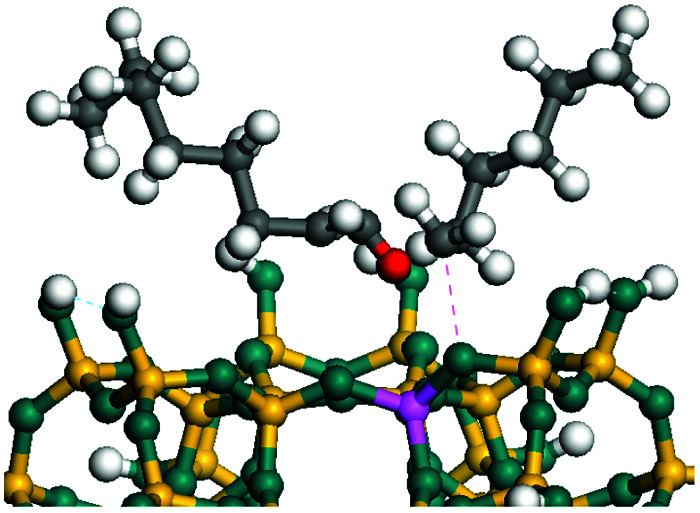	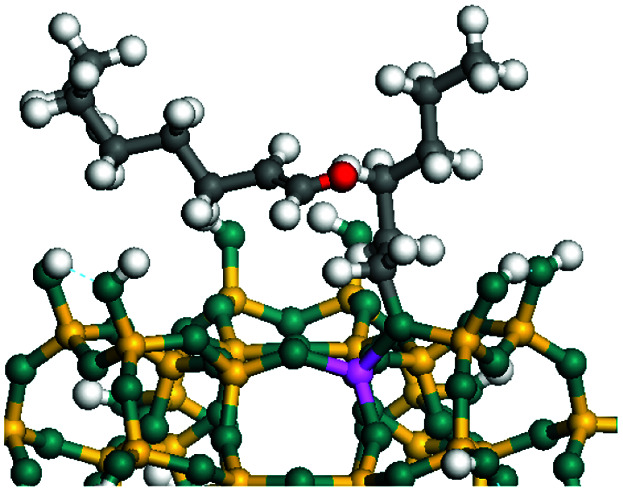	+64.22	+175.96
Cracking reaction	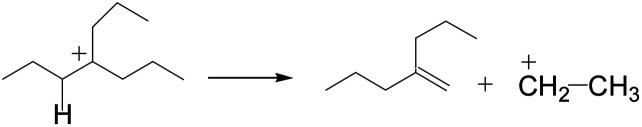			
Carbonium	Transition state	Reactant	Reaction energy kJ mol^−1^	Activation energy kJ mol^−1^
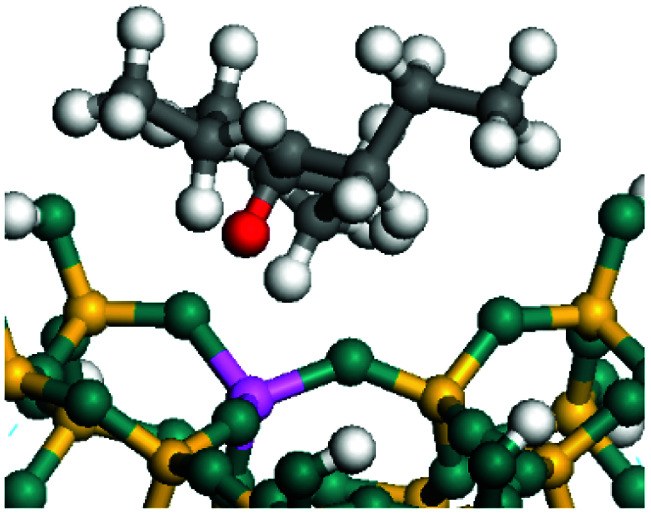	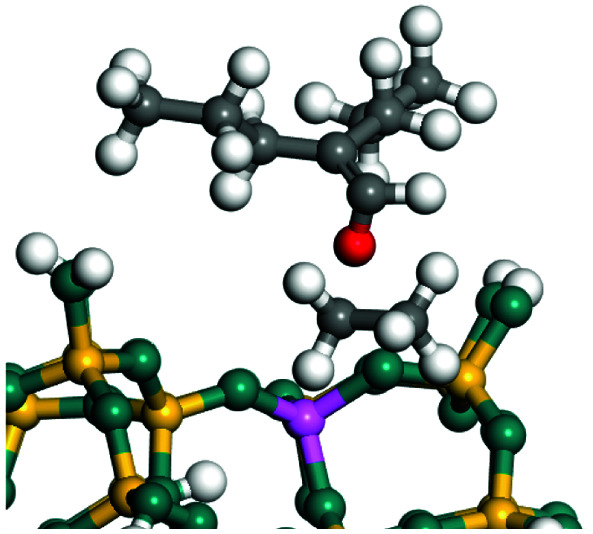	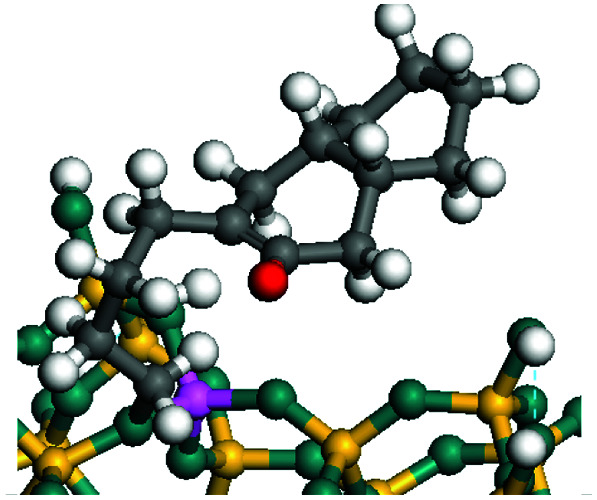	+89.86	+201.59
Cracking reaction	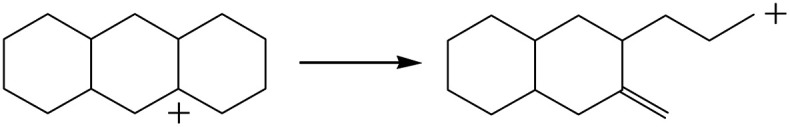			
Carbonium	Transition state	Reactant	Reaction energy kJ mol^−1^	Activation energy kJ mol^−1^
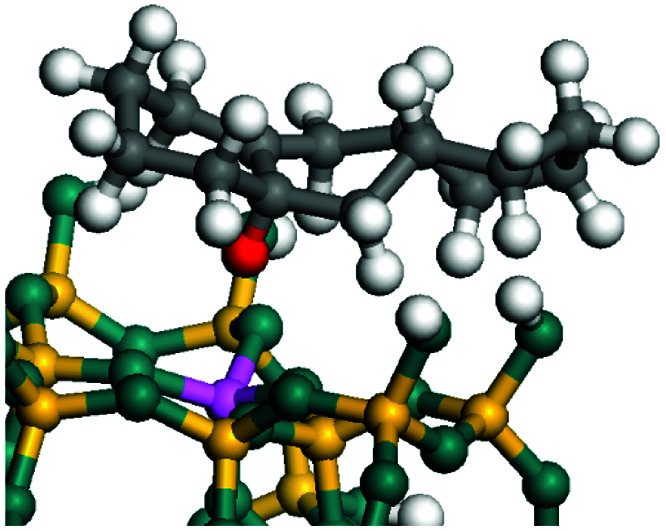	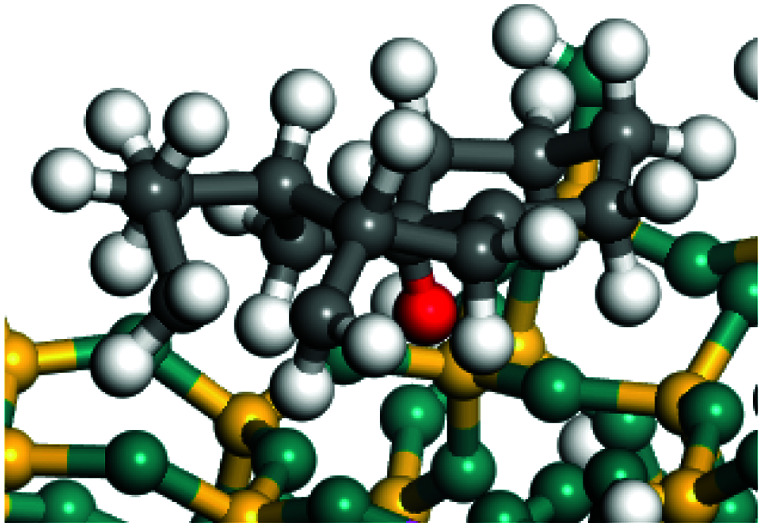	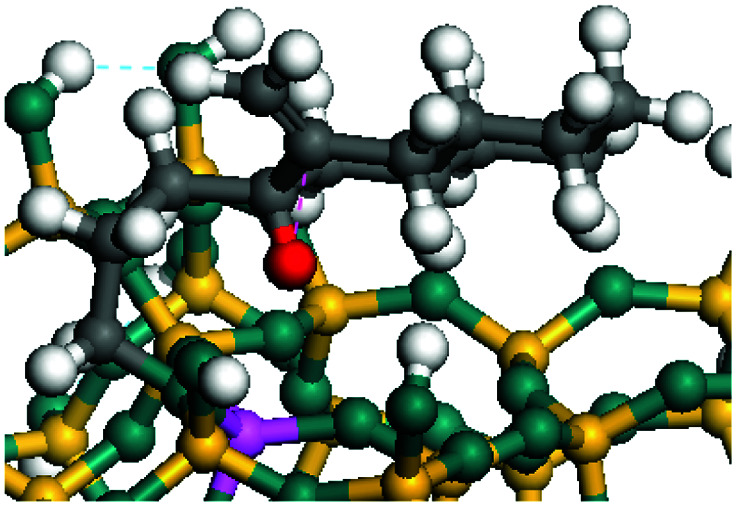	+63.71	+179.69

The cracking details of the protonated cycloalkanes on FAU-Al are listed in Table 8. Different from the alkanes, cycloalkanes maintained the single molecular structure adsorbed on the zeolite surface during cracking; therefore, the cracking energy of cycloalkane was approximately 10 kJ mol^−1^ lower than those of the alkanes. The similarity of the cycloalkanes to the alkanes was that the molecular weight hardly affected the cracking energy. It should be noted that the cracking of cycloalkanes was a multipath process for the β-section could occur at the junction of the bridged ring or on one side of the naphthenic ring. The β-section on the bridged ring involved slightly lower reaction energy and activation energy than that on the naphthenic ring. The possible reason could be the greater number of substituent groups on cyclohexene. Based on the close activation energy values, it could be predicted that both of these pathways may be effective in cracking the cycloalkanes.

**Table 8:** Cracking of cycloalkenes on FAU-Al

Cracking route 1	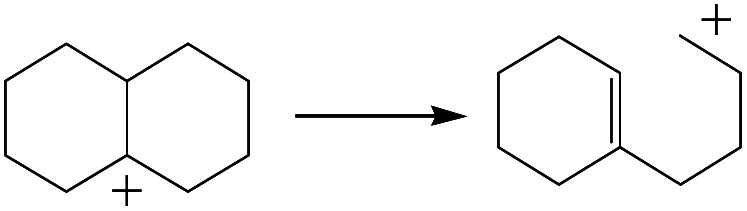			
Carbonium	Transition state	Reactant	Reaction energy kJ mol^−1^	Activation energy kJ mol^−1^
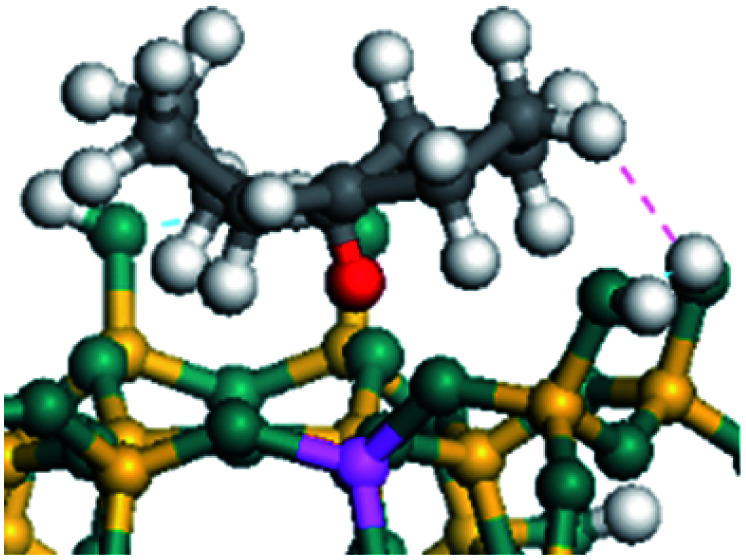	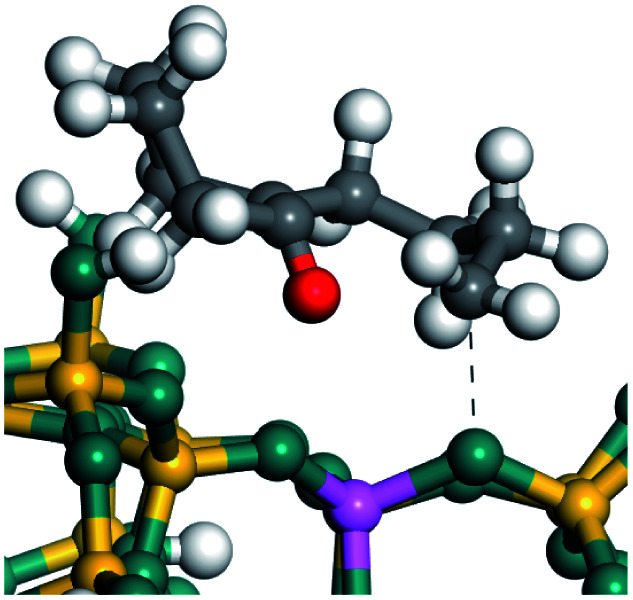	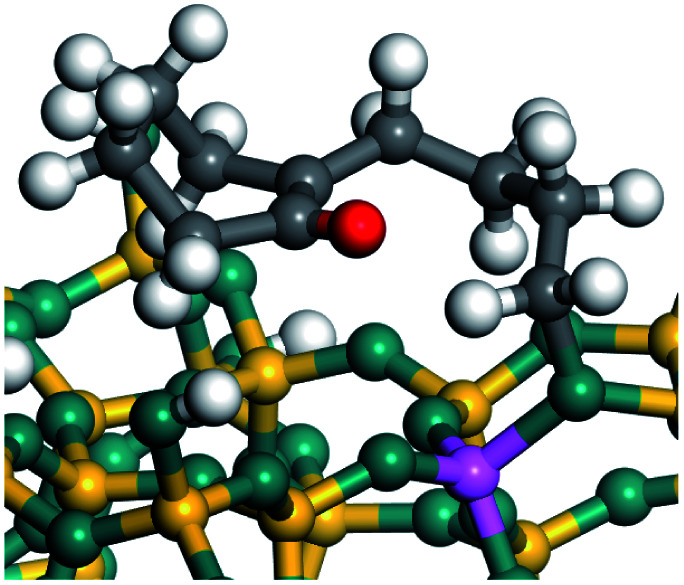	+50.54	+154.52
Cracking route 2	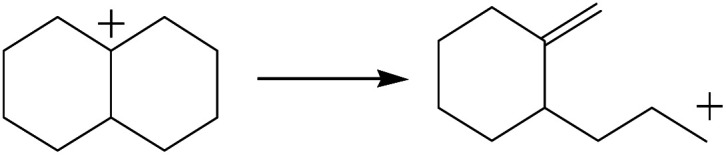			
Carbonium	Transition state	Reactant	Reaction energy kJ mol^−1^	Activation energy kJ mol^−1^
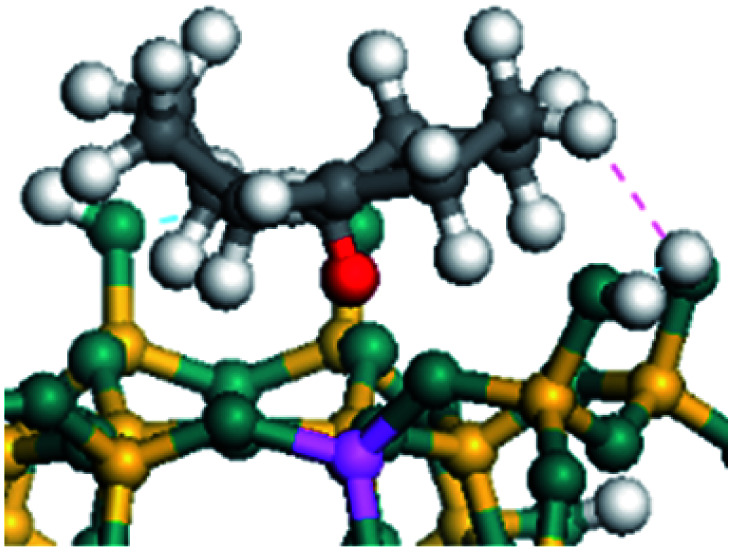	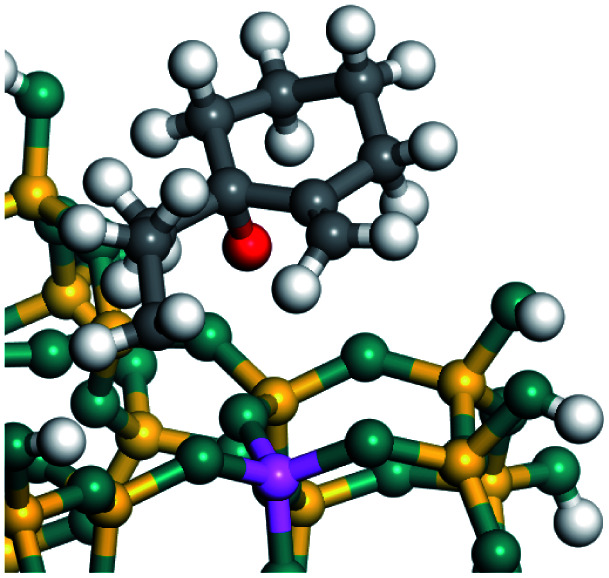	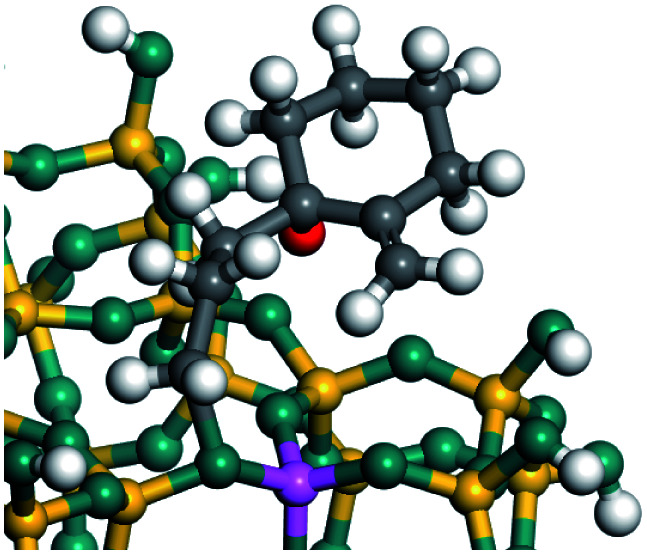	+56.87	+158.46
Cracking route 1	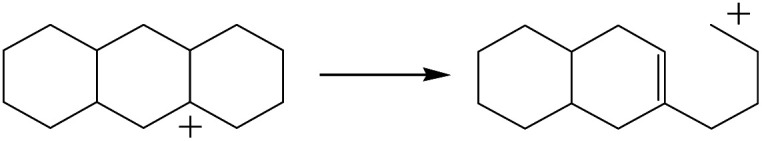			
Carbonium	Transition state	Reactant	Reaction energy kJ mol^−1^	Activation energy kJ mol^−1^
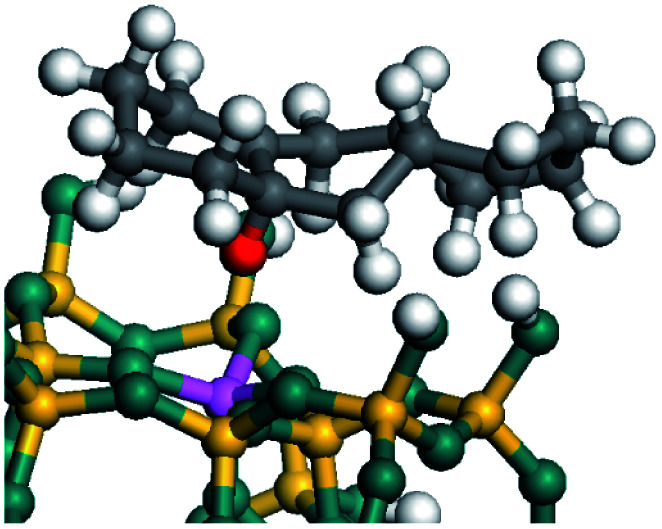	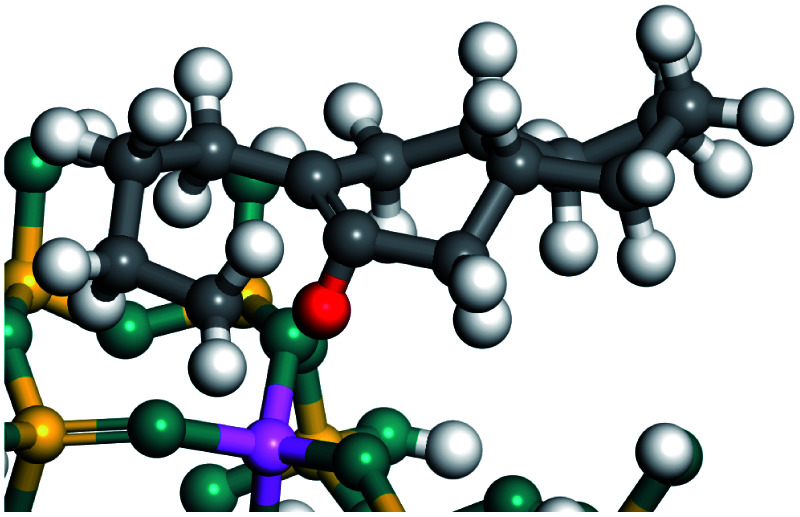	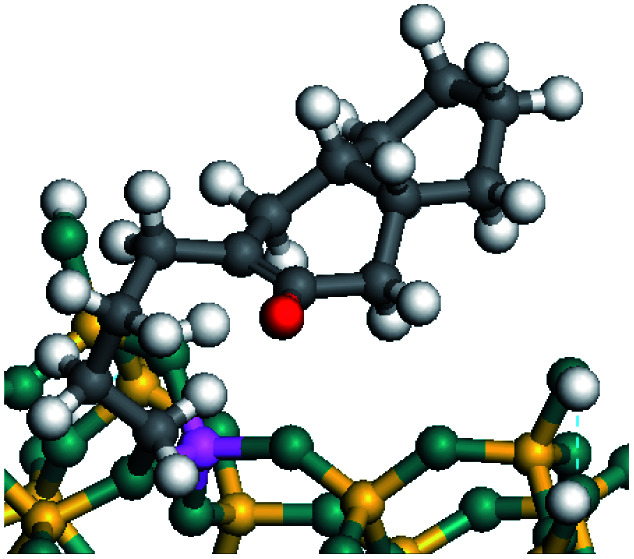	+52.91	+146.00
Cracking route 2	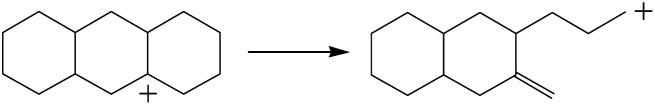			
Carbonium	Transition state	Reactant	Reaction energy kJ mol^−1^	Activation energy kJ mol^−1^
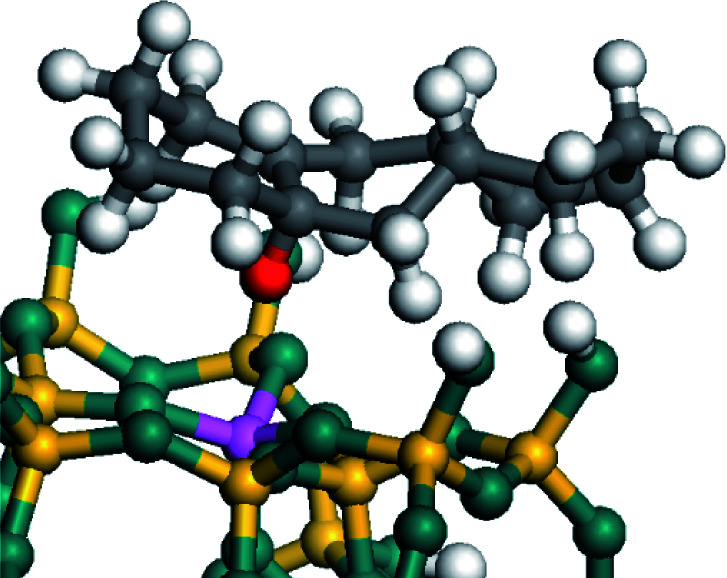	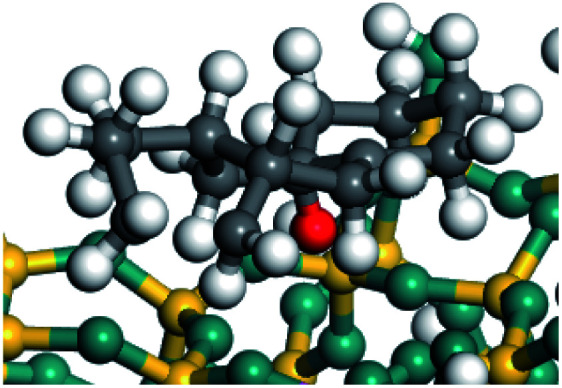	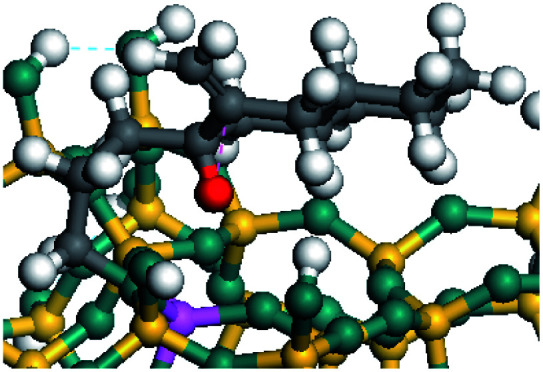	+57.25	+152.97

The cracking details of protonated tetrahydronaphthalene and tetrahydroanthracene are shown in Table 9. According to the calculation results, the stable structure of the reactant is an aromatic ring with a single long side chain. The carbonium was transferred to the end of the side chain and bonded with oxygen after cracking. Although the reaction paths of cycloalkyl-benzene and cycloalkanes are similar, the reaction energy and the activation energy of the aromatics were much lower than those of the cycloalkanes. The rebuild of the conjugated electrons effectively lowered the energy of the system, leading to a much lower reaction energy and activation energy. It can be concluded that once the aromatic ring is protonated, the cracking of the adjacent naphthenic ring would become relatively easier, and the aromatic ring can be treated as the energetic carrier of carbonium.

**Table 9:** Cracking of aromatics on FAU-Al

Cracking reaction	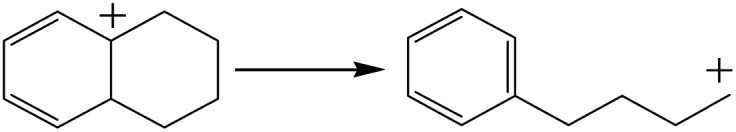				
Carbonium	Transition state	Reactant	Reaction energy kJ mol^−1^		Activation energy kJ mol^−1^
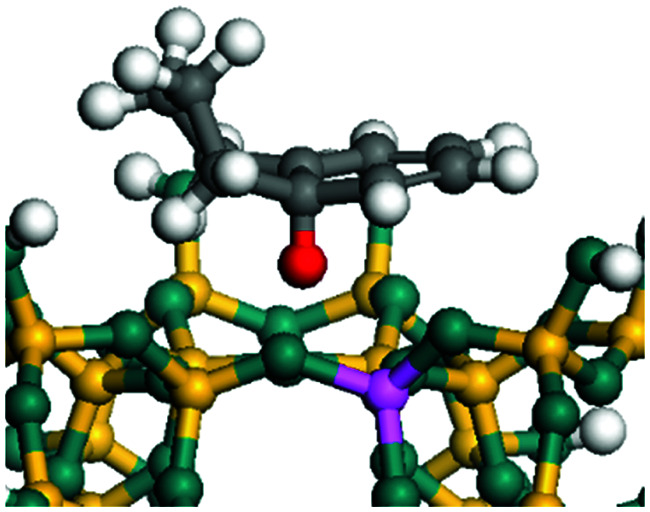	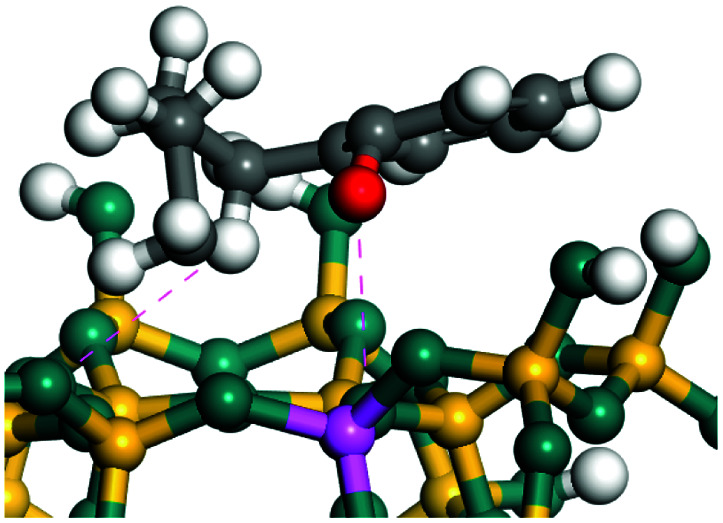	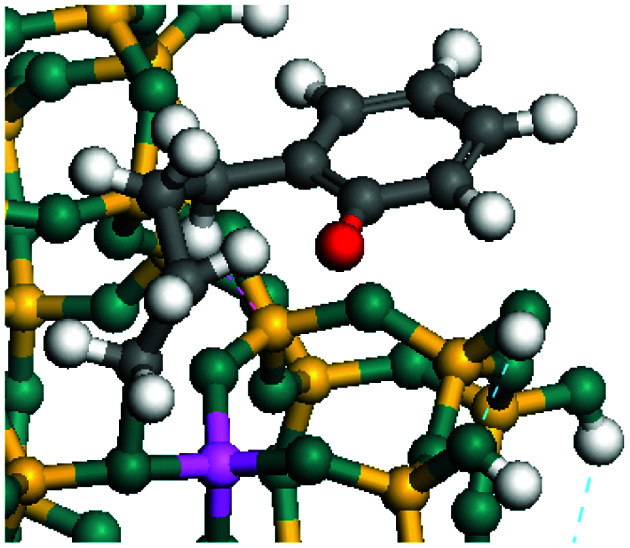	+20.96		+125.25
Cracking reaction	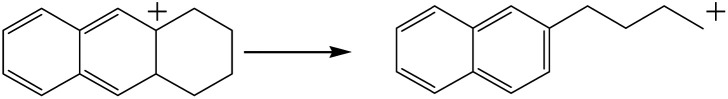				
Carbonium	Transition state	Reactant		Reaction energy kJ mol^−1^	Activation energy kJ mol^−1^
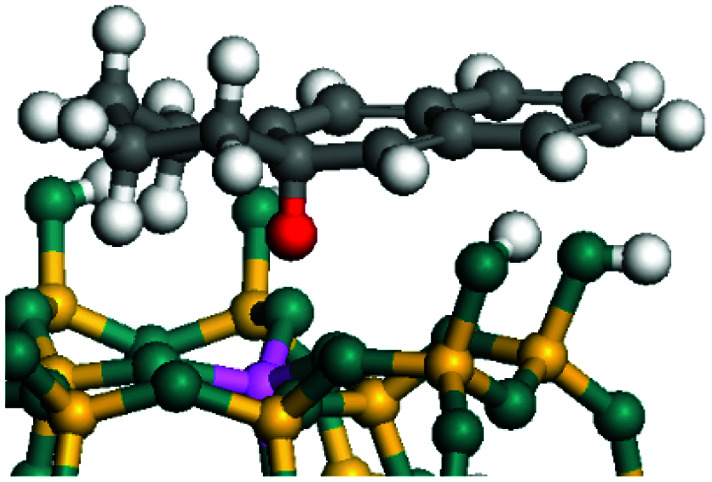	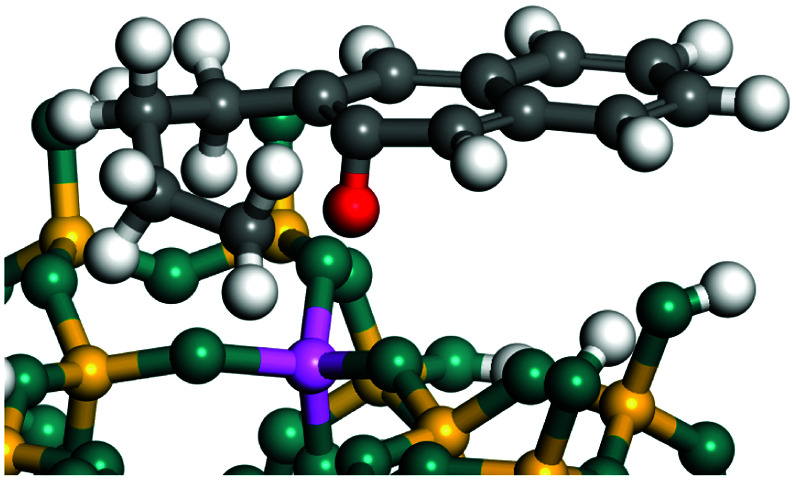	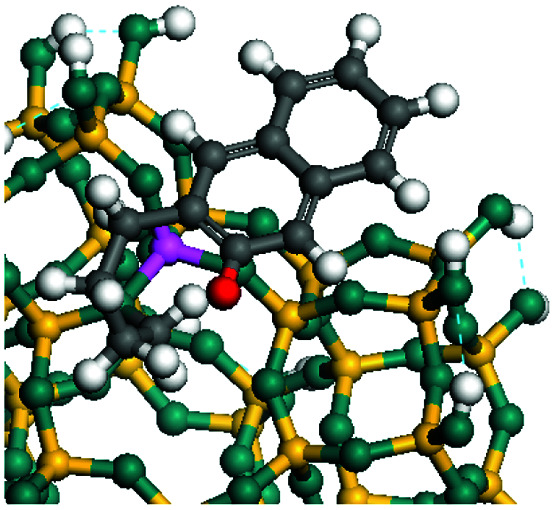		+22.20	+131.94

## 4 Conclusions

By evaluating the hydrocracking process of C-10 and C-14 alkanes, cycloalkanes and cycloalkyl-substituted aromatics, it could be concluded that the hydrocracking of alkanes and cycloalkanes involves at least three rate-controlling steps: dehydrogenation on the Ni–Mo–S active sites, the generation of carbonium, and the cracking of the carbon chain. The dehydrogenation of saturated hydrocarbons is a strongly endothermic reaction with very high activation energy. Meanwhile, this step is also restricted by the competitive adsorption of the aromatic rings and the reverse reaction trend under high hydrogen pressure. The protonation of olefins is relatively easier, whereas cracking saturated hydrocarbons with one carbonium is another rate-controlling step with high activation energy. In contrast, the hydrocracking route of cycloalkyl-substituted aromatics begins at the zeolite acid centers. The major rate-controlling step is the protonation of the aromatic ring. The effect of molecular weight on the intrinsic dynamics of the elementary reaction is limited, whereas the competitive adsorption ability of larger molecules is stronger than that of the smaller ones.

## Conflicts of interest

We declare that we have no financial and personal relationships with other people or organizations that can inappropriately influence our work. There is no professional or other personal interest of any nature or kind in any product, service and/or company that could be construed as influencing the position presented in, or the review of, the manuscript entitled.

## Supplementary Material
